# NANOS3 suppresses premature spermatogonial differentiation to expand progenitors and fine-tunes spermatogenesis in mice

**DOI:** 10.1242/bio.059146

**Published:** 2022-04-08

**Authors:** Hiroki Inoue, Takayuki Sakurai, Kazuteru Hasegawa, Atsushi Suzuki, Yumiko Saga

**Affiliations:** 1 Department of Gene Function and Phenomics, Mammalian Development Laboratory, National Institute of Genetics, Mishima, 411-8540 Japan; 2 Department of Genetics, School of Life Science, The Graduate University for Advised Studies (SOKENDAI), Mishima, 411-8540 Japan; 3 Division of Materials Science and Chemical Engineering, Faculty of Engineering, Yokohama National University, Yokohama, Kanagawa, 240-8501 Japan; 4 Department of Biological Sciences, Graduate School of Science, The University of Tokyo, Tokyo, 113-0033, Japan

**Keywords:** Nanos3, Spermatogenesis, Mouse, Retinoic acid, Testis

## Abstract

In the mouse testis, sperm originate from spermatogonial stem cells (SSCs). SSCs give rise to spermatogonial progenitors, which expand their population until entering the differentiation process that is precisely regulated by a fixed time-scaled program called the seminiferous cycle. Although this expansion process of progenitors is highly important, its regulatory mechanisms remain unclear. NANOS3 is an RNA-binding protein expressed in the progenitor population. We demonstrated that the conditional deletion of *Nanos3* at a later embryonic stage results in the reduction of spermatogonial progenitors in the postnatal testis. This reduction was associated with the premature differentiation of progenitors. Furthermore, this premature differentiation caused seminiferous stage disagreement between adjacent spermatogenic cells, which influenced spermatogenic epithelial cycles, leading to disruption of the later differentiation pathway. Our study suggests that NANOS3 plays an important role in timing progenitor expansion to adjust to the proper differentiation timing by blocking the retinoic acid (RA) signaling pathway.

## Abstract (precis)

**Summary:** This study demonstrated that NANOS3 functions as a timekeeper to prevent premature differentiation in order to initiate synchronized differentiation upon retinoic acid stimulation.

## INTRODUCTION

Spermatogenesis is a dynamic process that includes the proliferation and differentiation of spermatogonia, meiosis of spermatocytes, and elongation (spermiogenesis) of haploid spermatids. Many genes are complexly and precisely involved in these processes (Chen et al., 2018; Griswold, 2016; Matzuk and Lamb, 2008).

In mouse spermatogenesis, spermatogonial stem cells (SSCs) are maintained throughout life and continuously give rise to spermatogonial progenitors by incomplete cytokinesis (Greenbaum et al., 2011; Huckins and Oakberg, 1978; Iwamori et al., 2012). Due to incomplete cytokinesis, the number of connected cells in a cluster of spermatogonia approximately reflects the cell status. Single spermatogonia (A_single_, A_s_) and interconnected cells (A_paired_, A_pr_) mostly comprise the stem cell population, and clusters of more than three connected spermatogonia (A_aligned_, A_al_) comprise the progenitor population. All of these cells have stem cell properties and are termed ‘undifferentiated spermatogonia’ (Hara et al., 2014). Recent single cell transcriptome analyses followed by RNA velocity analyses revealed transition states in SSCs and progenitor spermatogonia, in which the activation of mTORC1 pathway was revealed to be a key step to initiate differentiation from SSCs (Suzuki et al., 2021b). This is consistent with our previous model that NANOS2, expressed mainly in As and Apr, represses mTORC1 signaling to maintain SSCs (Zhou et al., 2015). Undifferentiated spermatogonia initiate differentiation upon retinoic acid (RA) stimulation (Ikami et al., 2015; Lord et al., 2018; Suzuki et al., 2021a), after which progenitor cells express c-KIT and STRA8 (stimulated by RA 8) and differentiate into ‘differentiated spermatogonia’ (A_1_, A_2_, A_3_, A_4_, intermediate and B spermatogonia) (Griswold, 2016; Van Pelt and de Rooij, 1991). After spermatogonial differentiation, differentiated spermatogonia undergo exactly six rounds of mitosis before entering meiosis. The required time for cells to complete spermatogonial differentiation into spermatozoa is fixed for each species and may be variable among species (França et al., 1998; Griswold, 2016; Leblond and Clermont, 1952). The maintenance of the stem cell population and proliferation of the progenitor population are both important events for the production of the enormous number of spermatozoa in the testes throughout life. Although there are many studies investigating the mechanisms regulating stem cell maintenance (Helsel et al., 2017; Kanatsu-Shinohara and Shinohara, 2013; Kitadate et al., 2019; Lord et al., 2018; Lovelace et al., 2016; Murakami et al., 2020; Song et al., 2016; Suzuki et al., 2021b; Yoshida, 2019; Zhou et al., 2015), the mechanisms regulating progenitor function are mostly unknown.

NANOS is an RNA-binding protein that is evolutionarily conserved among many organisms and plays essential roles during germ cell development (Lehmann and Nusslein-Volhard, 1991; Kobayashi et al., 1996; Subramaniam and Seydoux, 1999; Wang and Lehmann, 1991). Three *Nanos* genes (*Nanos1*, *Nanos2* and *Nanos3*) were identified in the mouse, among which *Nanos2* and *Nanos3* are expressed specifically in germ cells (Tsuda et al., 2003). *Nanos2* is expressed in a male-specific manner and plays important roles in leading germ cells to male-type differentiation in the embryonic stage (Suzuki and Saga, 2008). NANOS2 is predominantly expressed in the stem cell population in the postnatal stage and postnatal *Nanos2*-deficiency results in the failure of stem cell maintenance (Sada et al., 2009; Zhou et al., 2015). Moreover, artificial induction of NANOS2 in spermatogonia inhibits their differentiation (Sada et al., 2009). NANOS2 interacts with mTOR and represses mTORC1 signaling by sequestering the mTOR complex to P-bodies, resulting in cell cycle arrest (Zhou et al., 2015). On the other hand, NANOS3 is expressed in both male and female germ cells in the embryonic stage. Postnatally, NANOS3 expression is mainly observed in A_aligned_, A_al_ spermatogonia, although low expression was observed in SSCs (Suzuki et al., 2009; Tsuda et al., 2003). The NANOS3 expression pattern, which is different from that of NANOS2, suggests that it has specific functions in spermatogonial progenitor cells. However, the NANOS3 function in spermatogenesis remains unclear because all germ cells lacking NANOS3 degenerate by embryonic day 12.5 (Suzuki et al., 2008; Tsuda et al., 2003).

In this study, to assess the role of NANOS3 in spermatogenesis, we established two distinct conditional knockout (cKO) mouse lines. Conditional deletion of *Nanos3* before birth resulted in the reduction of spermatogonial progenitor cells because of their premature differentiation without a notable influence on the spermatogonial stem cell population. We propose that a NANOS3-mediated mechanism functions in securing time for progenitor expansion and this is an important step to set up spermatogonial differentiation timing to maintain the precisely controlled seminiferous stages.

## RESULTS

### Generation of *Nanos3* conditional knockout mice

As *Nanos3*-deficient mice completely lose germ cells before birth (Suzuki et al., 2008; Tsuda et al., 2003), we established a conditional knockout mouse line to examine the function of *Nanos3* during spermatogenesis. As one of the strategies, we generated a bacterial artificial chromosome transgenic (BAC-Tg) mouse line expressing a floxed red fluorescent protein (RFP)*-*tagged NANOS3 (NANOS3-RFP) under the control of *Nanos3* regulatory elements (Fig. S1A). First, we confirmed that the transgene rescued the germ cell-loss phenotype in *Nanos3*-deficient mice (Fig. S1B–F). RFP signals were observed in a portion of the GFRA1-positive spermatogonial stem cell population and most NGN3-GFP-positive progenitor cells (Fig. S1G), whereas NANOS3-RFP was downregulated in KIT-positive cells (Fig. S1G). These observations suggested that NANOS3-RFP reproduces endogenous NANOS3 expression.

**Fig. 1.**
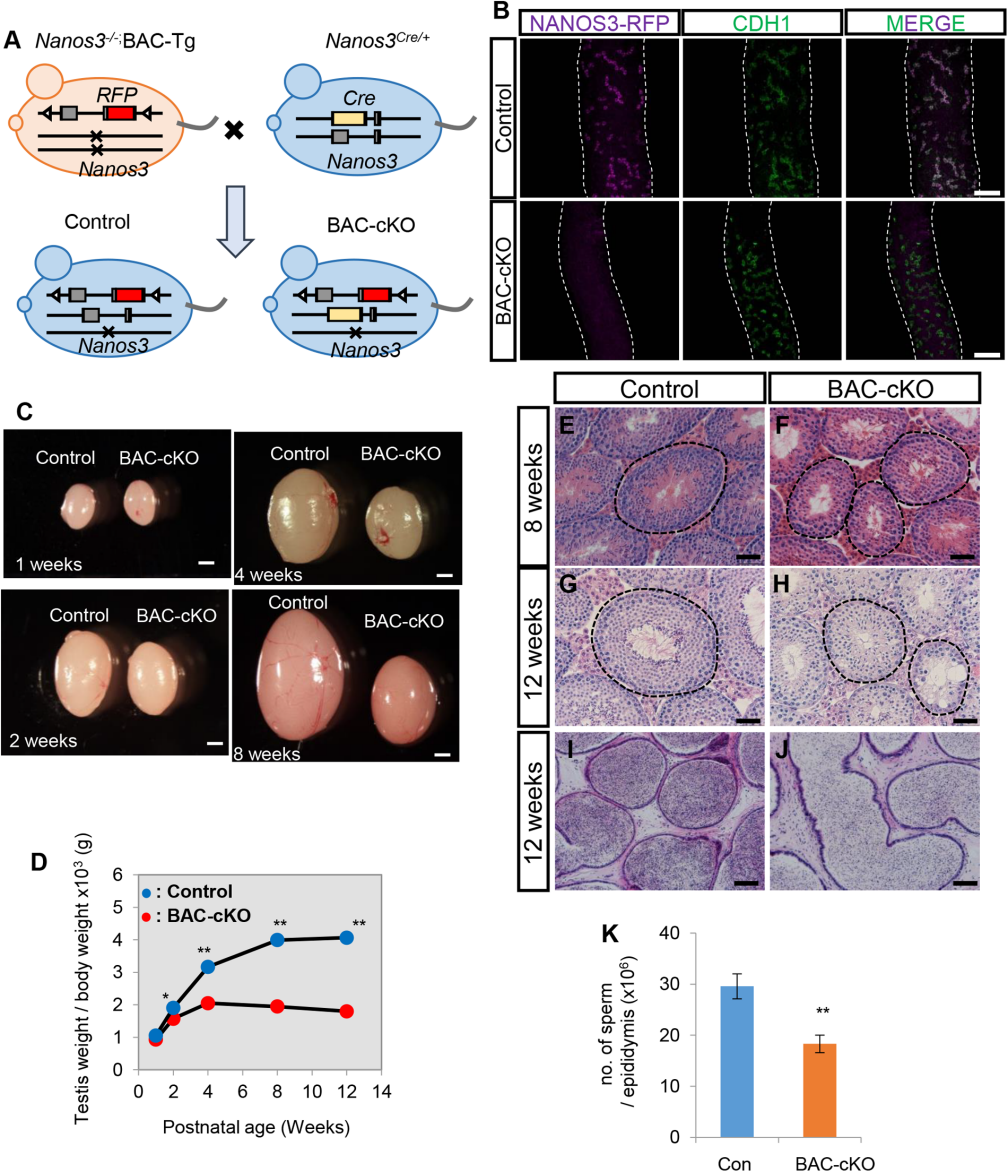
**Testicular abnormalities observed in BAC-cKO mice.** (A) Experimental scheme to obtain BAC-cKO males. *Nanos3*^−/−^; *BAC-Tg* females were crossed with *Nanos3^cre^*^/+^ males. The *Nanos3-Rfp* sequence is removed by *Nanos3-Cre* during germ cell development from E14.5. A *Nanos3^−/−^; BAC-Tg* male was used as the control. (B) Wholemount immunostaining of seminiferous tubules in 8-week-old testes. The signals of anti-RFP and anti-CDH1 are shown in magenta and green, respectively. The white dotted lines represent the outline of seminiferous tubules. Scale bars: 100 μm. (C) Testes from 1, 2, 4 or 8-week-old control and BAC- cKO mice. Scale bars: 1 mm. (D) Body and testis weights were measured in control and cKO mice at 1, 2, 4, 8 and 12 weeks of age. The testis weight was normalized by body weight. Values represent the mean±s.e.m. *P<0.05, **P<0.01. (E–J) Hematoxylin and Eosin (H&E)-stained cross-sections of control and mutant testes from 8- or 12-week-old mice (E–H) and epididymides from 12-week-old mice (I,J). Scale bars: 50 μm (E–H), 100 μm (I,J). (K) Counts of spermatozoa in a 12-week-old cauda epididymis (control: *n*=6, mutant: *n*=3). Values represent the mean±s.e.m. *P<0.05.

To eliminate *Nanos3-Rfp* during spermatogenesis, we used *Nanos3-Cre* mice (Suzuki et al., 2008). Although NANOS3 is expressed in primordial germ cells (PGCs) from embryonic day (E) 7.25 to E13.5 (Tsuda et al., 2003), the *Nanos3*-driven Cre recombinase activity was very low in PGCs but increased after E13.5 (Fig. S1I) (Suzuki et al., 2008), suggesting that this Cre line is useful to knockout *Nanos3-Rfp* at perinatal stages. To obtain BAC-*Nanos3* conditional knockout (BAC-cKO) mice, we crossed a *Nanos3−/−,* BAC-Tg female with a *Nanos3-*Cre/+ male (Fig. 1A). Wholemount immunostaining for RFP and CDH1 (E-cadherin), a marker of undifferentiated spermatogonia, revealed that almost all CDH1-positive cells in the BAC-cKO testes lacked NANOS3-RFP at 8 weeks (Fig. 1B). This confirmed that *Nanos3-Rfp* was deleted in undifferentiated spermatogonia.

Next, we examined temporal changes in the testicular size of BAC-cKO mice. The ratio of testis weight to body weight was smaller in the BAC-cKO than in control mice at 2 weeks and this difference was greater at later stages (Fig. 1C,D). On histological analyses, seminiferous tubules in BAC-cKO testes were smaller than those in control testes at 8 weeks and 12 weeks (Fig. 1E–J). However, although the number of spermatozoa in the cauda epididymis, the storage organ of functional sperm, and the testis weight significantly decreased in BAC-cKO mice (Fig. 1K), many spermatogenic cells and spermatozoa were still observed in the mutant testis and epididymis, respectively (Fig. 1E–J). We therefore examined whether these spermatozoa, which were produced without NANOS3, were functional. We crossed BAC-cKO males with wild-type females and assessed whether the *Nanos3-Rfp*-deleted allele was transmitted to the next generation (Fig. S2A). Unexpectedly, some offspring still had the undeleted *Nanos3-Rfp* gene. Moreover, some had both the deleted and undeleted sequence (Fig. S2B). This suggested that more than one copy of the BAC-transgene was integrated into a single BAC-Tg locus, and some progenitors escaped from *Nanos3*-KO (Fig. S2C). To verify the efficiency of *Nanos3*-deletion in more detail, we counted clones that escaped from deletion. Only 16.7% of clones still retained *Nanos3*-RFP at 4 weeks of age (Fig. S2D), which was much lower than the rate of progeny obtained by mating at later stages (6 out of 9). This suggests that most spermatogenic cells lacking *Nanos3* failed to become functional sperm and the escaped cells preferentially underwent normal spermatogenesis. However, we also obtained offspring derived from sperm with only the deleted transgenic allele (Fig. S2B). Thus, NANOS3 is dispensable for functional sperm production.

### Undifferentiated spermatogonia were reduced in BAC-cKO testes

**Fig. 2.**
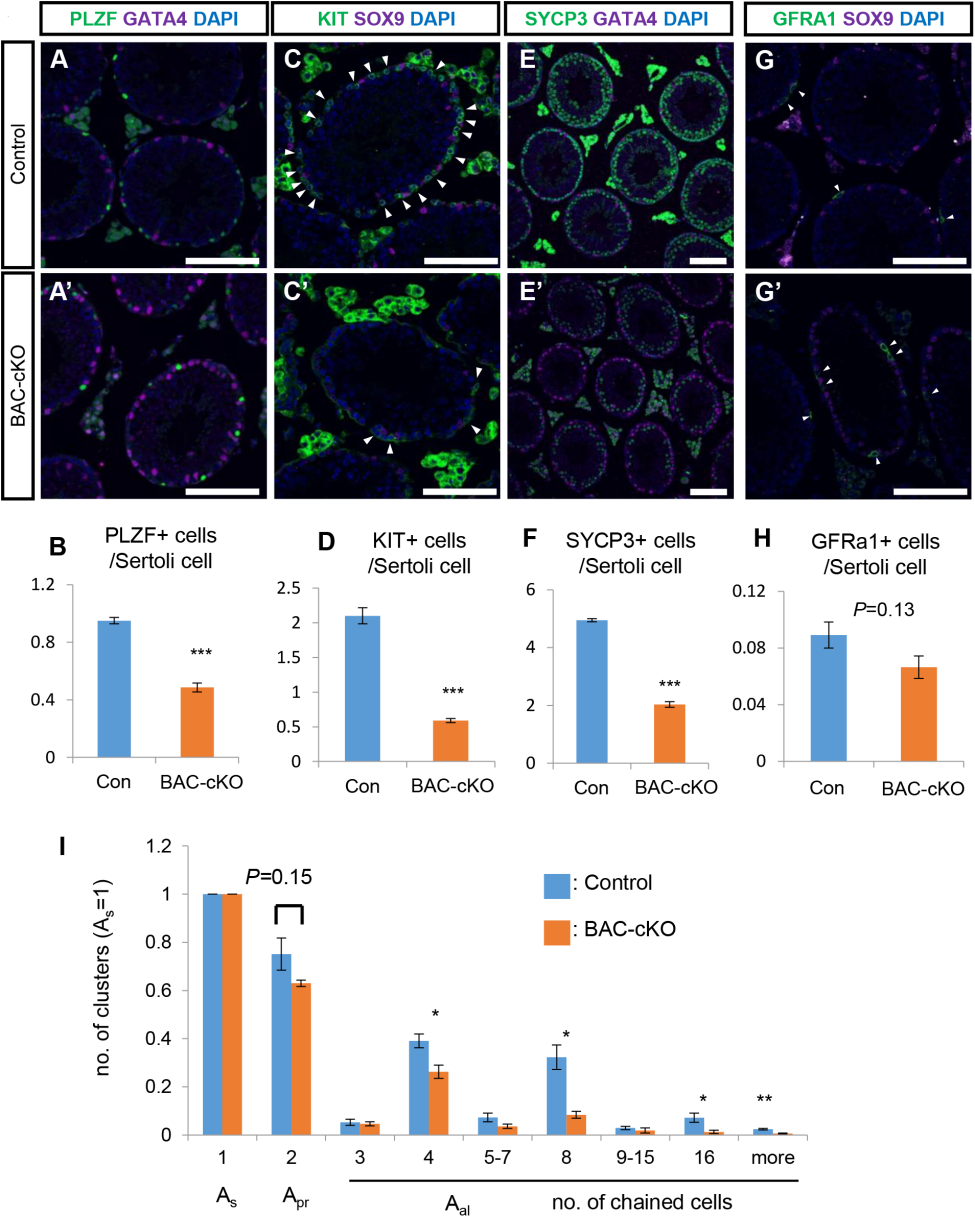
**Quantification of undifferentiated and differentiating spermatogonia in BAC-cKO mice.** (A–H) Immunostaining of 8-week-old testes with germ cell markers to distinguish developmental states; anti-GFRA1 (stem-state cells including population among undifferentiated spermatogonia, A,A′), anti-PLZF (undifferentiated spermatogonia, C,C′), anti-KIT (differentiated spermatogonia, E,E′) or anti-SYCP3 (spermatocytes, G,G′), and Sertoli cell markers, anti-GATA4 or anti-SOX9. The signals of germ cell markers are shown in green and signals for Sertoli cells are shown in magenta. Nuclei were counterstained with DAPI (blue). Scale bars: 100 μm. The number of germ cells was normalized by the number of Sertoli cells (B,D,F,H). Germ cell marker-positive cells are indicated by arrowheads. Values represent the mean±s.e.m. **P<0.001. (control: *n*=3, mutant: *n*=3). (I) Quantification of the clusters detected by wholemount immunofluorescence for CDH1. The horizontal axis represents the number of cells in a cluster. Cluster counts were normalized by the A_s_ spermatogonia number (*n*=3). Values represent the mean±s.e.m. *P<0.05, **P<0.01.

Although functional sperm were produced in cKO testes, the testis size was notably reduced in cKO mice (Fig. 1C,D). As NANOS3 is predominantly expressed in undifferentiated spermatogonia (Fig. S1G,H) (Suzuki et al., 2009), we performed immunostaining for PLZF, a marker of undifferentiated spermatogonia, using testis cross-sections to examine the number of PLZF-positive spermatogonia (Fig. 2A). The relative number of PLZF-positive undifferentiated spermatogonia in BAC-cKO testes was significantly lower than that in the control testis (Fig. 2A,B). Consistent with this reduction, the numbers of KIT (a marker of differentiating spermatogonia)-positive spermatogonia and SYCP3 (a marker of meiosis)-positive spermatocytes were lower in BAC-cKO testes (Fig. 2C–F). PLZF-positive cells contain the stem population in which GFRA1 is expressed. The number of GFRA1-positive spermatogonia was slightly reduced, but there was no significant difference between control and BAC*-*cKO testes (Fig. 2G,H), suggesting that the formation and maintenance of GFRA1-positive population was not affected by the depletion of NANOS3.

Spermatogonia expand their population by incomplete cell divisions and remain attached via intercellular bridges (Greenbaum et al., 2011; Huckins and Oakberg, 1978; Iwamori et al., 2012). We next examined which population of undifferentiated spermatogonia was affected by NANOS3 depletion. For this purpose, we performed wholemount immunostaining for CDH1 and RFP to count only *Nanos3*-deficient spermatogonia in BAC-cKO mice. The number of A_pr_ spermatogonia was not significantly different between control and mutant mice (Fig. 2I). However, the number of A_al4_ spermatogonia in the BAC-cKO was 33% less than that in the control. Moreover, A_al8-16_ and longer spermatogonial cluster counts were reduced by more than 70% in the BAC-cKO, suggesting that NANOS3 is required to expand A_al_ spermatogonia.

### Premature differentiation of undifferentiated spermatogonia occurs in the BAC-cKO testes

We hypothesized that the observed reduction of spermatogonial progenitors in BAC-cKO testes was caused by a cell proliferation defect and/or premature differentiation of spermatogonia. To test the former possibility, we performed immunostaining with anti-pH3, a marker of dividing cells (Fig. S3A), and measured the proportion of proliferating CDH1-positive undifferentiated spermatogonia. We found that the proportion was similar between control and BAC-cKO mice (Fig. S3B), suggesting that the loss of NANOS3 does not affect the proliferation of spermatogonia.

Next, we examined the latter possibility. It was previously reported that RA secreted from Sertoli cells is required for the initiation of spermatogonial differentiation, and the expression of STRA8, which is an RA responsive gene, is one of the indicators of RA signal pathway activity in spermatogonial differentiation (Endo et al., 2015; Raverdeau et al., 2012). We performed immunostaining for STRA8 and PLZF, and found that the proportion of STRA8-positive undifferentiating spermatogonia increased in BAC-cKO testes (Fig. S3C,D). This suggests that premature differentiation of undifferentiated spermatogonia occurs in BAC-cKO testes, which may be the main cause of the reduction of longer A_al_ spermatogonia progenitors.

### Endogenous *Nanos3* cKO has more severe spermatogenic defects

**Fig. 3.**
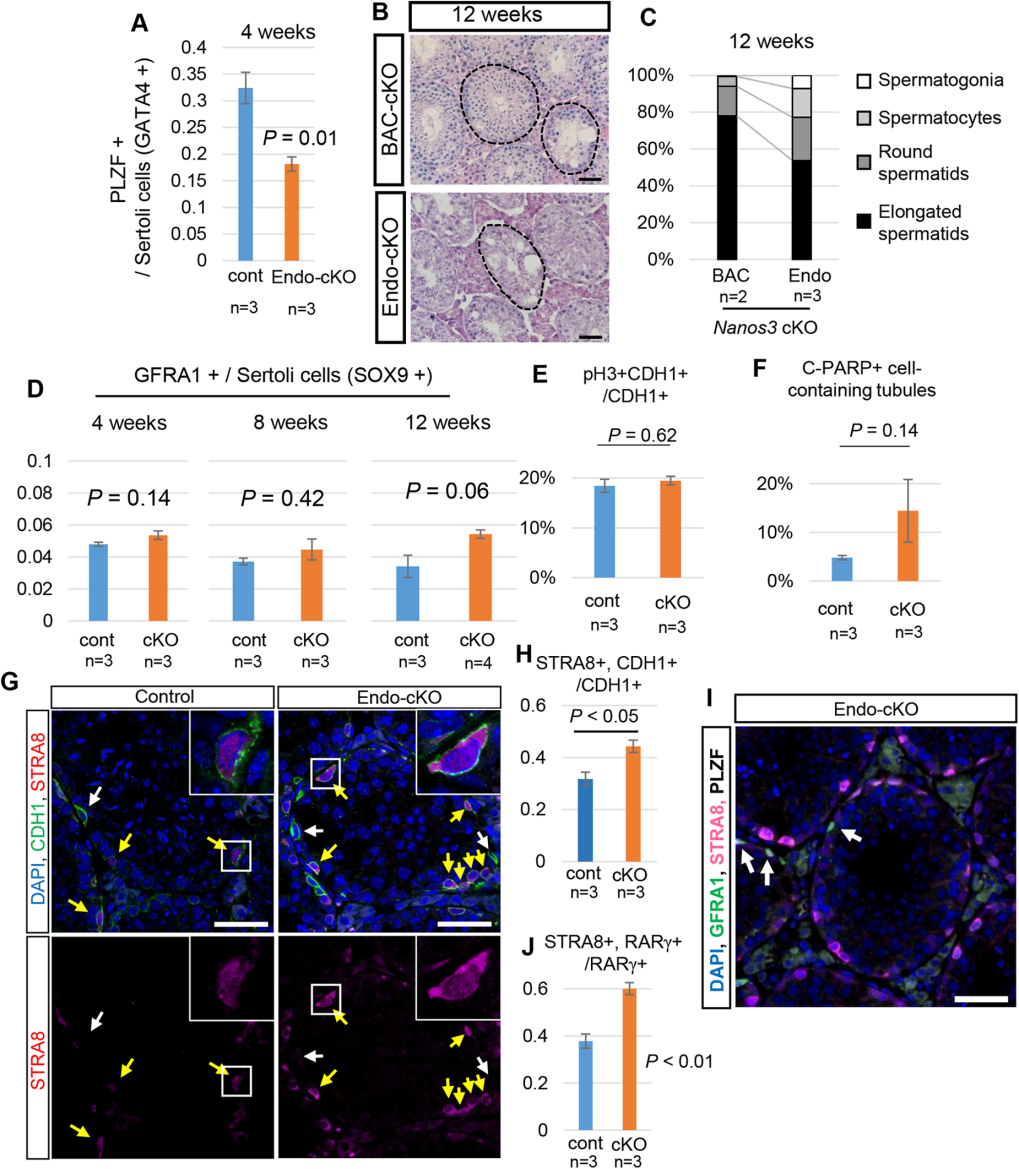
**Quantitative analyses of undifferentiated spermatogonia in endo-cKO mice.** (A) The number of PLZF-positive undifferentiated spermatogonia was normalized by the number of Sertoli cells (GATA4-positive cells). Values represent the mean±s.e.m. (B,C) Histological comparison between BAC-cKO and endo-cKO adult testes. H&E-stained cross-sections are shown (B). Scale bar: 100 μm. (C) The tubules were quantified based on the most differentiated spermatogenic cells (elongated, round-spermatids, spermatocytes or spermatogonia) contained in each tubule (BAC-cKO: *n*=2, endo-cKO: *n*=3). (D) The number of GFRA1-positive stem cells was normalized by the number of SOX9-positive Sertoli cells. Each cell type was counted by immunostaining sections of 4-, 8- and 12-week-old testes (*n*=3). Values represent the mean±s.e.m. (E) The ratio of pH3-positive proliferating spermatogonia to CDH1-positive undifferentiated spermatogonia in control (*n*=3) and endo-cKO (*n*=3). Values represent the mean±s.e.m. (F) The percentage of tubules that had c-PARP positive cells among counted tubules in control (*n*=3) and endo-cKO (*n*=3). Values represent the mean±s.e.m. (G) Immunostaining of 4-week-old testes with anti-STRA8 (red) and anti-CDH1 (green) antibodies. Nuclei were counterstained with DAPI (blue). Yellow arrows indicate STRA8-positive undifferentiating spermatogonia. White arrows indicate STRA8-negative undifferentiated spermatogonia. (H) The percentage of STRA8-positive cells among CDH1-positive undifferentiating spermatogonia. STRA8-positive cells significantly increased in endo-cKO testes. Values represent the mean±s.e.m. (I) Immunostaining of 4-week-old testes of endo-cKO mice with anti-GFRA1 (green), STRA8 (magenta) and PLZF (gray) antibodies. Nuclei were counterstained with DAPI (blue). GFRA1 and PLZF-double-positive stem cells indicated by arrows did not have STRA8 signals. Scale bar: 100 μm. (J) The percentage of STRA8-positive cells among the RARγ-positive spermatogonial progenitor population. STRA8-positive cells significantly increased in endo-cKO testes. Values represent the mean±s.e.m.

As discussed in the previous section (Fig. S2B), although small, an unignorable number of spermatogenic cells escaped from Cre recombinase in the BAC-cKO mice. Therefore, it is possible that some defects caused by NANOS3 loss are masked by the presence of normal germ cells retaining NANOS3. We therefore generated another *Nanos3-*cKO mice line in which exon 1 of endogenous *Nanos3* is floxed and deleted the exon by *Nanos3-Cre* (we referred to this line as endo-cKO) (Fig. S4A). The reduction in testis weight was comparable with that in the BAC-cKO line (Fig. S4B). Histological analysis also revealed that the number of spermatogenic cells progressively decreased with age in the endo-cKO testis. Although the diameter of the testicular tubules was similar to that in the control (Fig. S4C), the number of undifferentiated spermatogonia decreased (Fig. 3A; Fig. S4D), demonstrating that germ cell reduction started by 4 weeks in the endo-cKO. The testicular tubules became thinner than those in control testes at 8 weeks. At 12 weeks, there were many empty tubules in the endo-cKO testis, which was a more severe phenotype than observed for BAC-cKO, in which spermatids were still observed in many tubules at 12 weeks (Fig. 3B,C). Although spermatogenic cells were markedly affected, the GFRA1-expressing stem cell population and SOX9-positive Sertoli cells were observed in the 12 weeks endo-cKO testis (Fig. S4E). Moreover, the number of GFRA1-positive stem cells was maintained throughout all three time points (Fig. 3D). Thus, the GFRA1-positive population was maintained without cells escaping from Cre recombination, which may have occurred in the BAC-cKO, and confirmed that NANOS3 is dispensable for GFRA1-positive population maintenance.

### Premature differentiation also occurred in the endo-cKO

In the endo-cKO testis, spermatogenic cells were affected more markedly than in the BAC-cKO, suggesting additional defects. As germ cell reduction was already observed at 4 weeks in endo-KO (Fig. 3A), we used the 4 weeks testes for further analyses. The numbers of pH3-positive proliferating undifferentiated spermatogonia and cleaved-PARP (c-PARP)-positive apoptotic spermatogonia in the endo-cKO were not different from those in control testes (Fig. 3E,F; Fig. S4F–I), similar to the BAC-cKO (Fig. S3B). The number of c-PARP-positive tubules slightly increased in endo-cKO compared with that in the control (Fig. 3F; Fig. S4F,G). However, all c-PARP-positive cells were differentiated cells such as spermatocytes or round spermatids. As NANOS3 expression is repressed soon after spermatogonial differentiation (Fig. S1H), this increase in apoptotic cells may have been a secondary defect. Thus, apoptosis was not the cause of progenitor reduction. Similar to the BAC-cKO, the number of STRA8-positive spermatogonia significantly increased in endo-cKO testes (Fig. 3G,H). STRA8 expression was not obsNano's in the GFRA1-positive stem cell population (Fig. 3I) but observed specifically in the RARγ-positive progenitor population (Fig. 3J). These results confirmed the premature differentiation in spermatogonial progenitor cells among NANOS3-deficient spermatogenic cells.

### Disagreement in spermatogenic cell associations in the endo-cKO testis

**Fig. 4.**
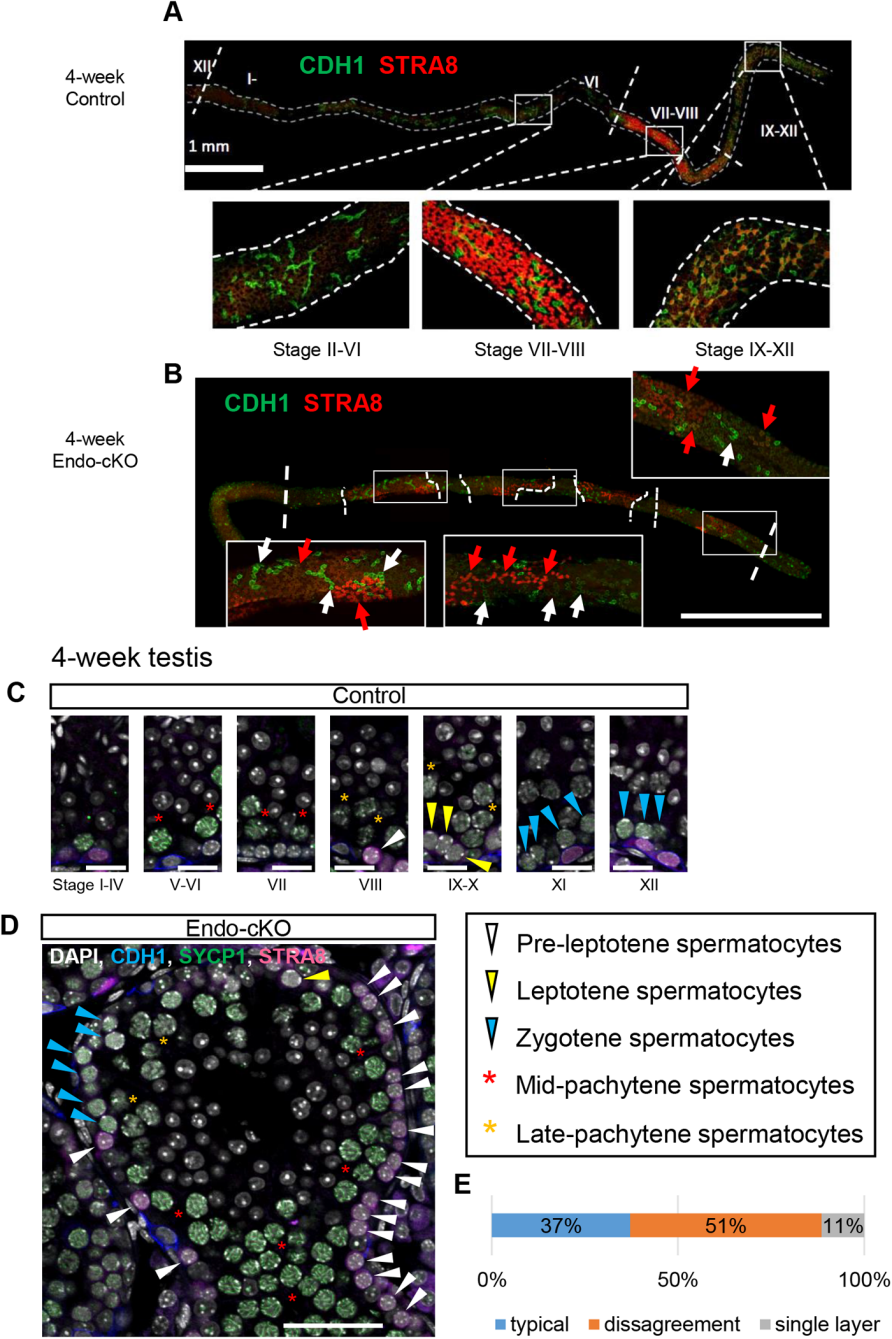
**Asynchronous STRA8 upregulation during spermatogenesis in the endo-cKO.** (A,B) The spliced images of multiple wholemount immunostaining images of testicular tubules from 4-week-old control (A) and endo-cKO mice (B). STRA8 and CDH1 signals are shown in red and green, respectively. The boundary of different STRA8 expression patterns is indicated by dashed lines. Bottom panels show the standard expression pattern of each domain. In the endo-cKO, there were intermittent STRA8-positive regions. STRA8-positive (indicated by red arrows) and -negative (indicated by white arrows) A_al_ clusters were intermingled. (C,D) Immunostaining of 4-week-old testes with anti-CDH1, anti-STRA8 and anti-SYCP1, which is a marker of zygotene to mid-pachytene spermatocytes. Nuclei were counterstained with DAPI (gray). Scale bar: 20 μm (C) or 50 μm (D). (E) Seminiferous stage distribution in endo-cKO mice. Seminiferous tubules were classified into three types: the tubules exhibiting typical cell associations (typical), those containing disagreeing cells (disagreement) and those having only a single layer (single layer).

In the mouse testis, spermatogonial differentiation and meiotic entry are precisely regulated by periodic RA stimulation and the resulting the RA signal pathway activation in a seminiferous stage-specific manner known as seminiferous cycles (Endo et al., 2015, 2017). Within tubules, stereotypical associations of germ cells, which are characterized by 12 distinct cellular association stages numbered I to XII, are observed in mice (Russell et al., 1990). The RA signal pathway is activated in undifferentiated spermatogonia at stages VII-XII and in spermatocytes at stages VII-VIII in mouse testes (Mark et al., 2015). We found that the number of STRA8-positive undifferentiating spermatogonia increased in the endo-cKO (Fig. 3G,H). This may have been caused by the following two scenarios: the stage-independent activation of STRA8 in undifferentiating spermatogonia or the disruption of the epithelial cycle duration. To distinguish these possibilities, we performed whole-mount immunostaining for STRA8 and CDH1. In rodents, the seminiferous tubule stages are arranged in order lengthwise along the tubules (Nakata, 2019; Nakata et al., 2017). The stage duration is well correlated with length of stages (Oakberg, 1956). Most STRA8-positive cells were confined to the expected stage VII-VIII-region and some were observed in the stage IX-XII-region in the control tubules (Fig. 4A), whereas in the endo-cKO, STRA8-positive cells were not restricted to a particular location and were interspersed along the tubule (Fig. 4B), suggesting that the stage dependency was disturbed in the absence of NANOS3, which may have resulted in the disagreement of cell association within a tubule. Thus, we next assessed this possibility by examining cellular combinations in each section. We classified spermatogenic cells into each differentiation step using several markers, i.e. CDH1, STRA8, and SYCP1, and the morphology of nuclei stained with DAPI. In the control, no atypical associations of spermatogenic cells were observed and all tubule sections were able to be classified into the seven stereotypical seminiferous stages (Fig. 4C; Fig. S5B; 169 tubules, *n*=3). However, in the endo-cKO testis, spermatogenic cells classified into different seminiferous stages were intermingled in a single tubule (Fig. 4D; Fig. S5B). This seminiferous stage disagreement was observed in approximately half of the tubules in the endo-cKO (Fig. 4E), although 37% of tubules in the endo-cKO exhibited typical seminiferous stages and all seven stages were observed (Fig. S5C). Moreover, GATA1, a stage-specific marker of Sertoli cells at stages VII–XII (Ketola et al., 2002; Matson et al., 2010; Yomogida et al., 1994), was strongly detected in almost all tubules in endo-cKO testes (Fig. S5A). This suggests that NANOS3 deletion caused premature differentiation through responding to the RA signal pathway, which disturbed developmental timing in both germ cells and Sertoli cells.

### Premature differentiation was suppressed in the absence of RA in endo-cKO

**Fig. 5.**
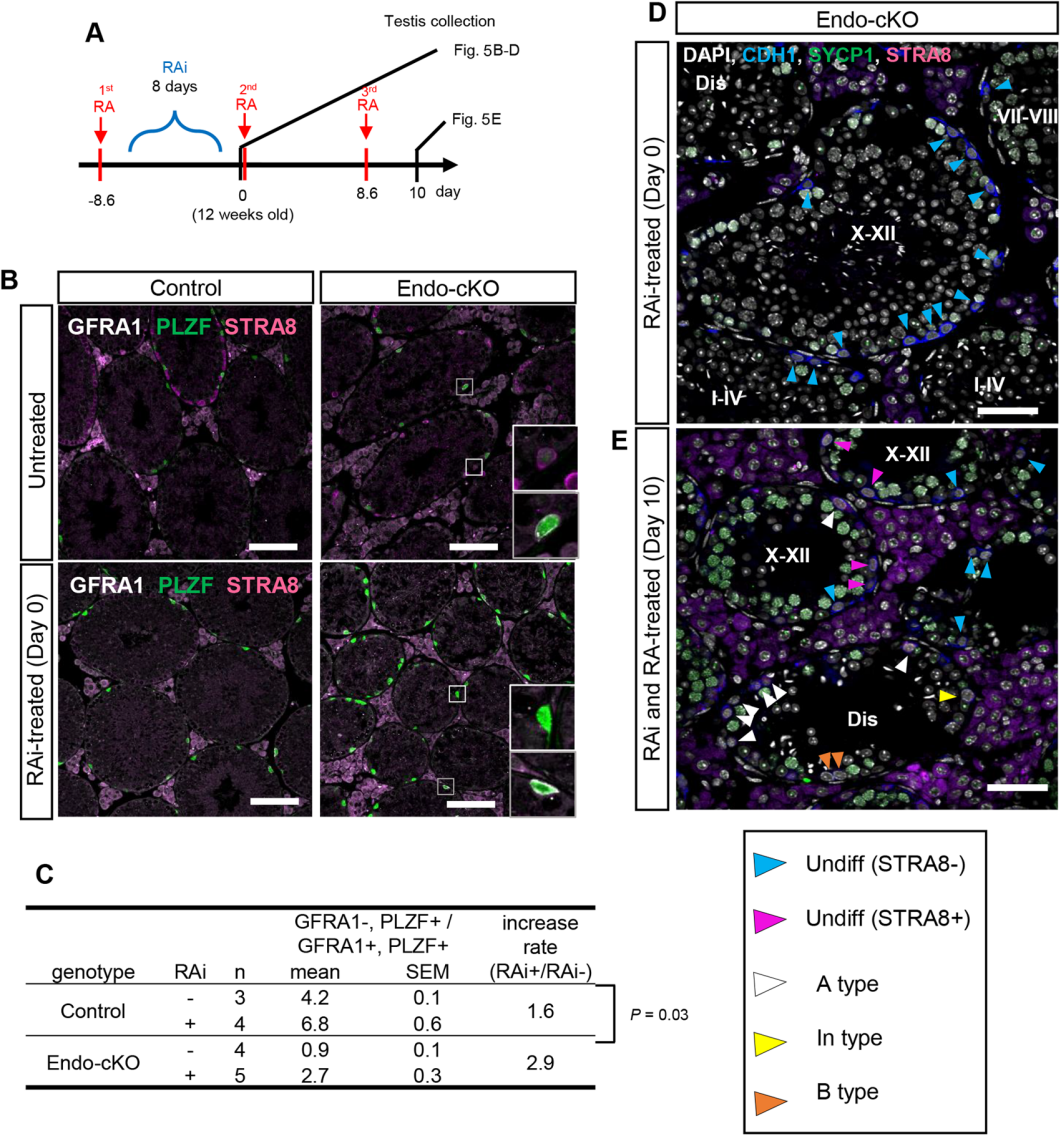
**The progenitor number was partially recovered by RA signal inhibition.** (A) Scheme of the experiment. RA was injected 8.6 days before 12 weeks of age, followed by daily RAi injection for 8 days. One of two testes was collected on the day of 12 weeks of age and then RA was injected again. At 1, 5 or 10 days or 5 days after the second RA injection, the other testis was collected. (B) Immunostaining of the RA-Rai-treated and untreated testes of control and endo-cKO. GFRA1, PLZF and STRA8 are shown in white, green and magenta, respectively. Scale bar: 100 μm. (C) The actual number and rate of increase of progenitors (GFRA1^−^, PLZF^+^) per stem cell (GFRA1^+^, PLZF^+^) are shown. (D,E) Immunostaining of testes at day 0 or 10. CDH1, SYCP1 and STRA8 are shown in blue, green and magenta, respectively. Nuclei were counterstained with DAPI (gray). Seminiferous stages are indicated in Roman numerals. Tubules containing atypical combinations of spermatogenic cells are labeled as ‘Dis’ (Disagreement). Scale bar: 50 μm.

We found premature differentiation as the likely cause of the NANOS3-null phenotype; reduction of the progenitor population of spermatogonia and disagreement of spermatocytes within a tubule. The spermatogonial differentiation of progenitor cells, which have RA receptors, can be induced by RA stimulation (Ikami et al., 2015; Suzuki et al., 2021a). If RA is involved in the premature differentiation in endo-cKO, its inhibition may rescue these phenotypes. To test this possibility, we treated mice with a RA synthesis inhibitor (RAi; WIN 18,446). We expected the number of progenitors to increase by inhibiting RA signaling. To evaluate the number of newly generated progenitors from stem cells within one seminiferous cycle (8.6 days), we initially injected RA and induced all progenitors to differentiate. Subsequently, we administered the RA inhibitor continuously for 8 days. The next day, we collected testes and evaluated the number of the progenitors that were newly produced during RA inhibitor treatment (Fig. 5A; Fig. S6A). As expected, STRA8 expression was repressed by RAi treatment even in the endo-cKO, whereas nearly all progenitor cells expressed STRA8 in untreated endo-cKO testes (Fig. 5B). This suggests that STRA8 upregulation depends on the RA signals even in the endo-cKO. The number of progenitors (GFRA1-negative, PLZF-positive cells) per GFRA1-positive undifferentiated spermatogonia (GFRA1-positive, PLZF-positive cells) in the endo-cKO increased by up to 2.9-times after RA and RAi treatment (0.9±0.1 versus 2.7±0.3; mean±SE) (Fig. 5C), but this ratio was only 1.6-times higher in the control after treatment (4.2±0.1 versus 6.8±0.6; mean±s.e.) (Fig. 5C), although the actual number of progenitors per GFRA1-positive spermatogonia was still less than that in the control even after RAi treatment (2.7±0.3 versus 6.8±0.6; mean±s.e.). The increasing ratio of progenitors normalized by the GFRA-positive spermatogonia was significantly higher in endo-cKO than in the control (Fig. 5C). Thus, the progenitor reduction in endo-cKO testes was rescued by the inhibition of the RA signaling pathway. Therefore, we considered the premature differentiation of progenitors by responding to RA to be a cause of progenitor reduction.

### The time required for spermatogonial differentiation once initiated does not change even in the endo-cKO

To further examine whether premature differentiation is a cause of the seminiferous stage disagreement in the endo-cKO, we assessed the seminiferous stage and spermatogenic cell distribution after synchronizing the seminiferous stage by RA administration after RAi treatment for 8 days (Fig. 5A; Fig. S6A). As mentioned above, progenitors accumulated in the testicular tubules at day 0, just before RA administration (Fig. 5E; Table S1). At this time point, there were no differentiating spermatogonia, or intermediate or type B spermatogonia, suggesting that spermatogonial differentiation was suppressed in both wild-type and endo-cKO testes (Table S2). One day after RA injection, which induced spermatogonial differentiation, all progenitors expressed STRA8 in both cases (Fig. S6B). The germ cells differentiated into intermediate and type B at day 5 (Fig. S6C). At 10 days after RA injection, these differentiating spermatogonia developed to zygotene spermatocytes in the control testis. Zygotene spermatocytes were included in most tubules (97%; 132 of 136 tubules), whereas leptotene and pre-leptotene spermatocytes or mid-pachytene spermatocytes were hardly observed in the day 10 endo-cKO testis (*n*=1). This suggests that germ cells that started differentiation at the same timing developed to at least the zygotene stage in a synchronized manner even in the absence of NANOS3. We also investigated the second round of spermatogonial differentiation in which the RA signal was no longer suppressed (Fig. 5A). Most tubules exhibited putative stages X-XII in the day 10 endo-cKO testis because 97% of tubules had zygotene spermatocytes. However, in approximately 20% of tubules, B type and/or intermediate spermatogonia co-existed with A type spermatogonia and zygotene spermatocytes (Fig. 5E; Table S2), demonstrating that stage disagreement was induced in the endo-cKO after stopping RAi treatment. These results suggest that the premature differentiation observed in endo-cKO resulted in the seminiferous stage disagreement. However, the seminiferous stage in the adult tubules was not synchronized completely in both control and KO (Table S1). Therefore, we cannot exclude the possibility that the unsynchronized cells induced the unsynchronized timing of spermatogonial differentiation because preleptotene spermatocytes and pachytene spermatocytes also produce RA and this RA production is resistant to WIN18,446 (Beedle et al., 2019; Endo et al., 2017; Raverdeau et al., 2012).

**Fig. 6.**
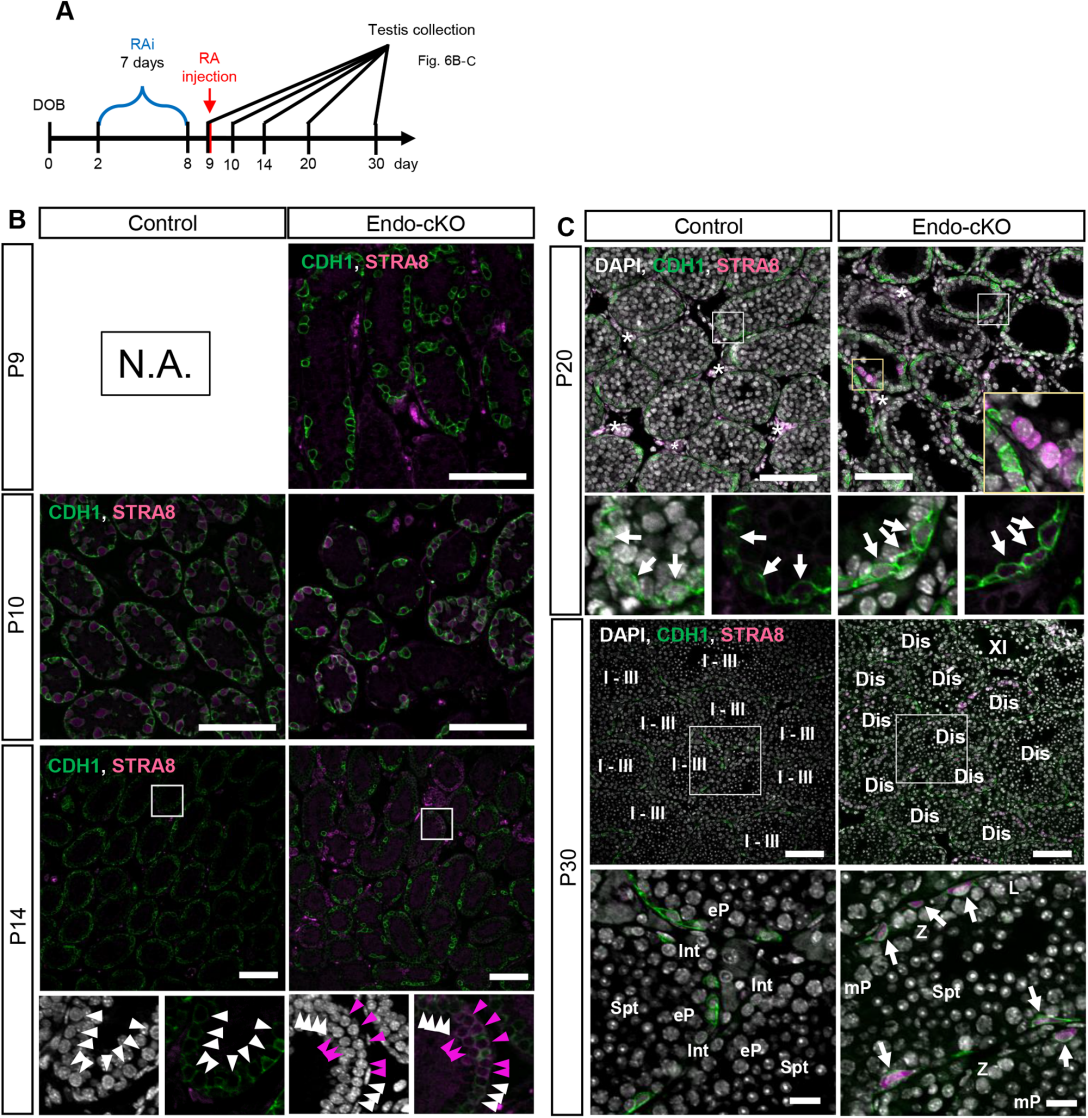
**Stage disagreement occurred even in the synchronized testicular tubules.** (A) Scheme of the experiment. RAi was daily injected for 7 days from P2 to P8, and then RA was injected. The testes were collected just before RA injection (P9), 1, 5, 11 and 21 days after RA (P10, P14, P20 and P30). (B,C) Immunostaining of testes at P9 to P30. CDH1 and STRA8 are shown in green and magenta, respectively. Nuclei were counterstained with DAPI (gray). (B) White and magenta arrowheads indicate STRA8-negative and -positive intermediate spermatogonia, respectively. (C) White arrows indicate undifferentiated spermatogonia. Spermatogenic stages are indicated by Int (intermediate spermatogonia), L (leptotene spermatocytes), Z (zygotene spermatocytes), eP (early pachytene spermatocytes), mP (mid or late spermatocytes) and Spt (round spermatids). Seminiferous stages are indicated in Roman numerals. Tubules containing atypical combinations of spermatogenic cells are labeled as ‘Dis’ (Disagreement). Scale bars: 100 μm in B and C, or 20 μm in magnified images in C. Asterisks indicate non-specific interstitial somatic signal.

To overcome this possibility, we also synchronized the seminiferous tubule stage using the WIN7D+RA method in neonatal mice (Fig. 6A; Hogarth et al., 2013; Beetle et al., 2019; Endo et al., 2017). With this method, 7-day RAi treatment from post-natal day 2 (P2) to P8 followed by RA injection at P9 can induce completely synchronized spermatogenesis. In the P9 testes (before RA injection), we confirmed that no germ cells, i.e. undifferentiated spermatogonia, expressed STRA8 in both control and cKO testes (Fig. 6B). This result is consistent with adult experiments (Fig. 5B).

We then investigated whether the synchronized differentiation is induced upon RA injection and how long it is maintained in the absence of NANOS3. In the control testes, the synchronized differentiation was confirmed at 30 days (P30: 21 days after RA injection), whereas different stages of tubules were observed in the cKO testes (Fig. 6C), indicating that the stage disagreement was already induced in the absence of NANOS3. To identify when this disagreement is induced, we next examined an earlier stage at P14 and P20. At P14, 5 days after RA exposure, germ cells are expected to become intermediate spermatogonia. As expected, we observed intermediate spermatogonia in both control and KO testes (Fig. 6B). However, some of the intermediate spermatogonia co-expressed STRA8 only in the cKO testes, indicating the failure of STRA8 downregulation. At P20 when no RA activity is expected (Endo et al., 2017), we detected STRA8-positive preleptotene spermatocytes and differentiating spermatogonia co-expressing STRA8 and CDH1 in cKO testes, which were never observed in the control testes (Fig. 6C), indicating that the stage disagreement in the progenitors already started at P20 (11 days after the first RA treatment) under this experimental condition. Therefore, stage disagreement was induced even in the synchronized tubules due to the absence of NANOS3.

### NANOS3 does not bind the known NANOS2-target mRNAs

**Fig. 7.**
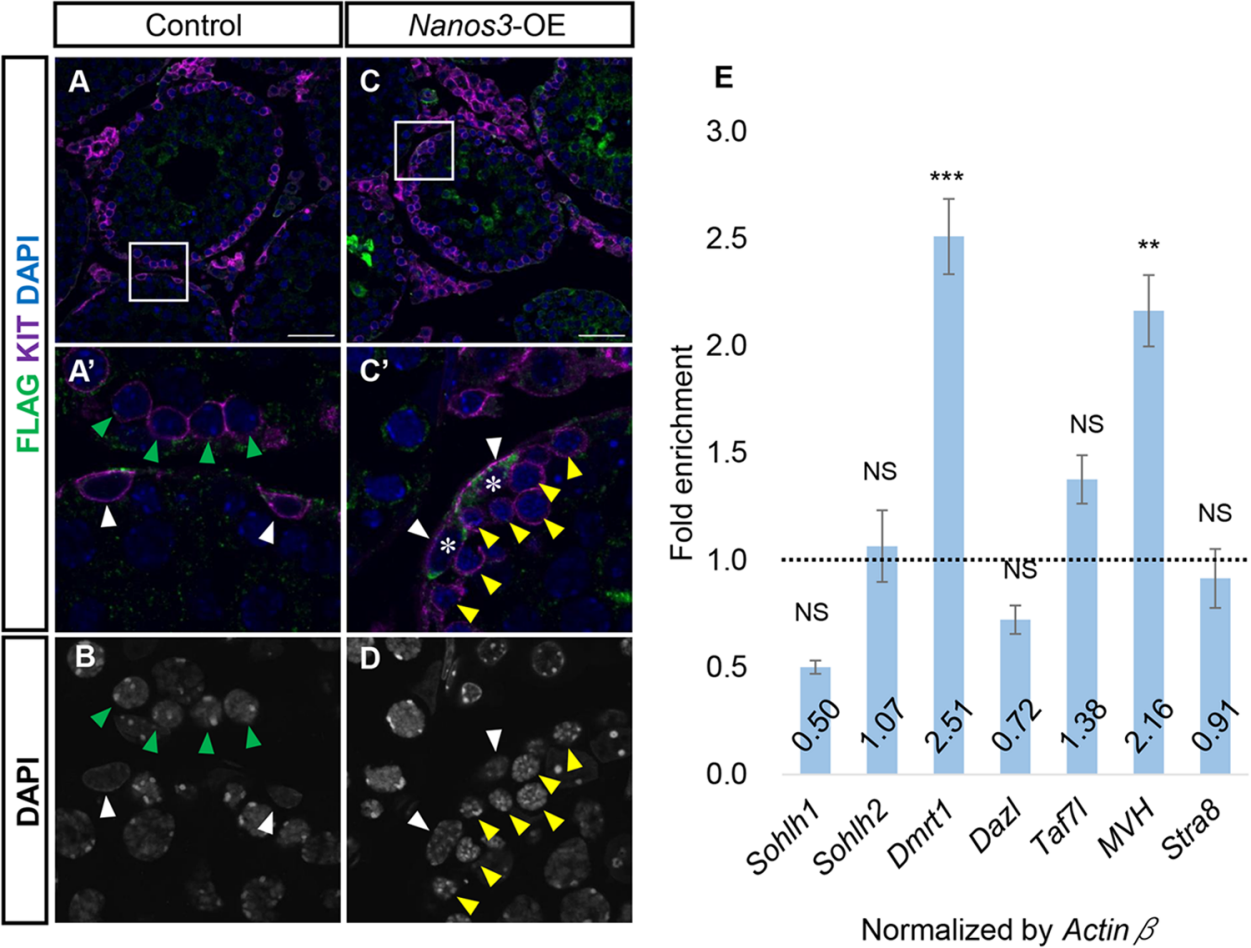
**NANOS3 target mRNAs differ from those of NANOS2.** (A–D) Immunostaining of 4-week-old testes with anti-FLAG-M2 (green) and anti-KIT (magenta) antibodies. Nuclei were counterstained with DAPI (blue). Magnified images of epididymides are shown in A′ and C′. Scale bars: 50 μm. White, green and yellow arrowheads indicate type A spermatogonia, type B spermatogonia and pre-leptotene spermatocytes, respectively. Asterisks indicate FLAG-positive spermatogonia. (E) RT-qPCR analyses of mRNAs co-precipitated with anti-FLAG antibody from testis extracts of 4-week-old Flag-tagged *Nanos3*-OE mice. The fold enrichment of each mRNA in IP of anti-FLAG compared with IP of IgG was calculated (ratio of each mRNA level in FLAG IP to IgG IP). *Actinβ* was used to normalize the mRNA enrichment level. Values represent the mean±s.d. **P<0.01. ***P<0.001.

Lastly, to gain insight into the molecular functions of NANOS3, we generated a *Nanos3*-overexpression (OE) mouse line that carries the *CAG-floxed-mRFP-3xFlag-tagged Nanos3* transgene (Fig. S7A) and performed NANOS3 RNA immunoprecipitation followed by quantitative-PCR (RIP-qPCR). To induce *3xFlag-tagged Nanos3* (*3xFlag-Nanos3*) expression in the male germ line, we used *Nanos3-Cre*. We confirmed the upregulation of *Nanos3* mRNA and the expression of 3xFLAG-NANOS3 protein in *Nanos3*-OE testes (Fig. 7A–D; Fig. S7B–E). Although the *3xFlag-Nanos3* transgene was driven by the CAG promoter, NANOS3 expression was restricted to undifferentiated spermatogonia and early-stage differentiated spermatogonia (Fig. 7A–D; Fig. S7D,E). This expression pattern was similar to that of endogenous NANOS3. However, abnormal cell clumps were observed in the lumen of seminiferous tubules in the *Nanos3*-OE testis and abnormal round cells instead of spermatozoa were observed in the *Nanos3*-OE epididymis (Fig. S7G–J).

Our previous studies revealed that the RNA-binding protein NANOS2 binds mRNAs of spermatogonia differentiation-related genes, such as *Sohlh1/2*, and represses their translation during spermatogenesis (Niimi et al., 2019; Zhou et al., 2015). These NANOS2-target genes are expressed in differentiating spermatogonia in which 3xFLAG-NANOS3 was detected. Therefore, to examine whether NANOS3 also bound the NANOS2-target mRNAs, we first immunoprecipitated Flag-tagged NANOS3 from testis extracts of the *Nanos3*-OE mice with anti-Flag antibody or control IgG and confirmed the precipitation of 3xFLAG-NANOS3 (Fig. S7K). As NANOS2 associates with mRNAs of *Sohlh1*, *Sohlh2*, *Dmrt1*, *Taf7l*, *Dazl* and *Stra8* genes, but not *Mvh* (*Ddx4*) (Niimi et al., 2019; Zhou et al., 2015), we next analyzed these mRNAs by immunoprecipitation of 3xFLAG-NANOS3 followed by RT-qPCR (Fig. 7E). Among the NANOS2-interacting mRNAs, only *Dmrt1* mRNA was significantly enriched by RNA-IP, although *Mvh* mRNA was unexpectedly co-precipitated (Fig. 7E), suggesting that NANOS3 targets are distinct from those of NANOS2 in spermatogenesis.

We then investigated the effects of the loss of NANOS3 on DMRT1 protein expression. DMRT1 is a transcription factor expressed in both germ cells (from GFRA1-positive spermatogonia to differentiated spermatogonia) and Sertoli cells (Matson et al., 2010). DMRT1 activates *Sohlh1/2* and represses *Stra8* to promote spermatogonial differentiation (Matson et al., 2010; Murphy et al., 2010). On immunostaining, DMRT1 was detected in most PLZF-positive undifferentiated spermatogonia in both wild-type and endo-cKO, and STRA8 expression was restricted to DMRT1-positive cells (Table S3). To evaluate the protein expression level of DMRT1 in undifferentiated spermatogonia, we quantified its signal intensity. Although the expression pattern was not different between wild-type and endo-cKO testes (Fig. S8A, Table S3), the relative signal intensity of DMRT1, which was normalized by the DAPI signal, was lower in the endo-cKO testis, suggesting that the loss of NANOS3 resulted in the downregulation of DMRT1. However, as NANOS3 is expected to repress the expression of target mRNA (Suzuki et al., 2014; Wright et al., 2020), we do not consider *Dmrt1* mRNA to be a direct target of NANOS3. As DMRT1 binds the *Stra8* promoter region and represses its transcription, DMRT1 downregulation may be the one of the reasons why STRA8 was upregulated in *Nanos3*-cKO spermatogonia.

## DISCUSSION

The importance of NANOS3 for the survival of PGCs was previously reported (Suzuki et al., 2008; Tsuda et al., 2003). However, because NANOS3-deficient mice lost germ cells in the embryonic stage, its role in spermatogenesis has not been addressed. In this study, we addressed this issue by conducting loss-of-function analyses of NANOS3 in spermatogenic cells *in vivo* and propose that NANOS3 plays a crucial role in timing progenitor expansion by blocking the RA signal pathway in order to properly time differentiation.

### Escapers from *Nanos3* Cre attenuate the phenotype

In this study, we established two distinct *Nanos3*-cKO mouse lines. STRA8 upregulation was observed in both (Fig. S3C,D; Fig. 3G,H); however, the phenotype of the BAC-cKO was milder than that of endo-cKO (Fig. 3B,C). One reason for this difference may be the efficiency of *Nanos3* gene deletion. According to genotype analyses of offspring, BAC*-*cKO mice have at least two transgenes integrated into a single locus because we observed progeny containing both intact *Nanos3*-*RFP* and deleted transgenes at a rate of approximately 50% (Fig. S2B,C). Thus, the inefficient removal of both transgenes resulted in the production of many escapers retaining intact *Nanos3* in BAC-cKO mice. On the other hand, endo-cKO mice have only one copy of *Nanos3*, which can be removed more efficiently. As the escapers can undergo spermatogonial differentiation at the proper stage, the presence of an unignorable number of escapers in the BAC-cKO testis may have buffered the stage disruption, leading to sufficient sperm production.

### NANOS3 may alter the RA sensitivity of undifferentiated spermatogonia

Under the suppressive conditions of spermatogonial differentiation by RAi treatment, the number of progenitors increased in both control and endo-cKO testes (Fig. 5C,D). In the control, only the synchronizing effects contributed to the increase in progenitor cell number. In the endo-cKO, the actual number of progenitors per stem cell was still lower than that in the RAi-treated control (2.7±0.3 versus 6.8±0.6; mean±s.e.; Fig. 5D). However, the rate of increase in the endo-cKO was significantly higher than that in the control (Fig. 5C). We reasoned this to be because the increase in progenitor number in endo-cKO was not only due to the synchronizing effects, but also to the rescue effects of RAi treatment via the inhibition of premature differentiation. Therefore, we concluded that premature differentiation was repressed, and the number of progenitors was rescued in endo-cKO testes. This suggests that NANOS3 represses the RA signaling pathway in order to maintain the progenitor state. The premature differentiation may thus be due to the abnormal upregulation of RA or high responsiveness to RA in the absence of NANOS3. The continuous supply of a higher level of RA induces premature spermatogonial differentiation, leading to germ cell death (Hogarth et al., 2015; Snyder et al., 2011). However, in endo-cKO testes, although premature spermatogonial differentiation was induced, only a slight increase in apoptotic cells was observed (Fig. 3F; Fig. S4H,I). Moreover, RA is supplied by Sertoli cells, and preleptotene and pachytene spermatocytes (Endo et al., 2017; Raverdeau et al., 2012). As all of these cells do not express NANOS3, the former possibility is unlikely to be the primary defect caused by NANOS3 deletion. Therefore, NANOS3-deficient spermatogonial progenitors may sense the low level of RA. NANOS3 may play a role in regulating the sensitivity to RA in spermatogonial progenitors.

### Is stage disagreement caused by premature differentiation?

Seminiferous cycle disagreement was observed in endo-cKO testes (Fig. 4D), which we considered to have been caused by the premature differentiation of progenitors (Fig. 5E,F). The timing of each step of spermatogenesis is fixed and precisely regulated (França et al., 1998; Gewiss et al., 2019; Leblond and Clermont, 1952). Even in endo-cKO, this strictly regulated time course was conserved at least until the zygotene stage (Fig. 5F; Fig. S6B,C) (Ashley et al., 2004; Raverdeau et al., 2012). In addition, a previous report demonstrated that once spermatogonial differentiation is induced, the germ cells can produce RA cell-autonomously without RA supplied from Sertoli cells and develop into spermatozoa (Raverdeau et al., 2012). This supports our hypothesis that the stage disagreement within tubules resulted from the acceleration of differentiation timing during the undifferentiated spermatogonial stage, especially the A_al_ stage (Fig. S8). Moreover, a portion of the first wave of intermediate spermatogonia failed to downregulate STRA8 (Fig. 6B). This error was not observed in adult or 4W cKO testes (Fig. S6C; Fig. 3G), suggesting that the defect of STRA8 downregulation was the first wave-specific event. There are several differences between first wave and steady state spermatogenesis. The first wave spermatogenic cells progress from a prospermatogonial-like state into the spermatogenic differentiation pathway without going through the undifferentiated stage (McCarrey, 2017; Yoshida et al., 2006). Therefore, the RA response in the first wave spermatogenic cells may be different from that in steady state spermatogenic cells. The stage-specific germ cell associations and duration of the germ cell differentiation process are fixed in a species-dependent manner in mammalian spermatogenesis (França et al., 1998; Gewiss et al., 2019; Russell et al., 1990). Moreover, even under the high RA conditions, stage disagreement of germ cells was not observed because germ cells were quickly removed by apoptosis (Hogarth et al., 2015; Snyder et al., 2011). In some primate species, such as humans, multi-stage tubules are present, but germ cell associations are conserved within patch-like compartments (Luetjens et al., 2005; Wistuba et al., 2003). Thus, the disruption of germ cell associations observed in our endo-cKO is a unique phenotype and the causative phenomenon remains unclear. The discrepancy in seminiferous stage may disturb stage-specific ‘Sertoli cell-germ cell’ and ‘germ cell-germ cell’ signal interactions or the activation of surveillance systems such as meiotic checkpoints.

### Possible molecular function of NANOS3

NANOS3 is a NANOS family RNA-binding protein. NANOS2, which is a paralog of NANOS3, is predominantly expressed in the GFRA1-positive spermatogonial population and plays a role in stem cell maintenance by suppressing the genes involved in spermatogonial differentiation (Suzuki et al., 2009; Zhou et al., 2015). Although NANOS3 is also involved in the inhibition of spermatogonial differentiation, only *Dmrt1* mRNA was bound by NANOS3 among the possible NANOS2 targets (Fig. 7E). This suggests that NANOS3 represses spermatogonial differentiation through a different mechanism than NANOS2. NANOS2 interacts with its partner protein DND1 and the CNOT complex, which is involved in the deadenylation of target mRNA (Suzuki et al., 2010, 2012, 2014; Wright et al., 2020). Similar to NANOS2, NANOS3 also interacts with DND1 and the CNOT deadenylation complex, but its binding to the CNOT complex and deadenylase activity are weaker than those of NANOS2 (Suzuki et al., 2014; Wright et al., 2020). Thus, NANOS3 may weakly function in the repression of spermatogonial differentiation through a similar molecular mechanism to NANOS2. Indeed, NANOS3 has redundant biological function with NANOS2 because NANOS2 can rescue the phenotype of NANOS3 loss in both embryonic PGC development and the postnatal spermatogenesis stage (Suzuki et al., 2007). However, NANOS3 cannot rescue or compensate for the loss of NANOS2 in spermatogenesis (Suzuki et al., 2007). This one-way redundancy may be explained by the difference in target mRNAs between NANOS2 and NANOS3. *Dmrt1* mRNA, which is a target of NANOS2 and DND1, was immunoprecipitated by NANOS3 (Fig. 7E). On the other hand, *Mvh* mRNA, which was significantly enriched in NANOS3 RIP-qPCR (Fig. 7E), is not bound by NANOS2 or DND1 (Niimi et al., 2019; Zhou et al., 2015). This suggests that NANOS3 has interactors other than DND1. In addition, NANOS2 is unsuitable for proliferating spermatogonial progenitors because it represses the cell cycle via mTORC1 inhibition (Zhou et al., 2015). One study suggested that NANOS3 overexpression prolongs the G1 phase in spermatogonia (Lolicato et al., 2008). However, in our *Nanos3* cKO study, no cell proliferation defect was observed (Fig. S3A,B; Fig. 3E; Fig. S4F,G). Thus, NANOS3, but not NANOS2, may be important for repressing the premature differentiation of progenitors to maintain them without altering their cell cycle.

DMRT1 was downregulated based on immunostaining of endo-cKO (Fig. S8A,B). This suggested that NANOS3 positively regulated DMRT1. However, NANOS2 and DND1 also bind *Dmrt1* mRNA (Niimi et al., 2019; Zhou et al., 2015), and NANOS2 expression was retained GFRA1-positive undifferentiated spermatogonia in the endo-cKO testes (Fig. S8C), suggesting that NANOS2 compensates for the repression of DMRT1 expression. The loss of DMRT1 in the spermatogonial progenitors resulted in precocious meiotic entry (Matson et al., 2010), but precocious meiotic entry was not observed in the endo-cKO testis after synchronization by RA inhibitor treatment (Fig. 5E; Fig. S6C). However, as our data are insufficient to exclude the possible effects of DMRT1 upon the lack of NANOS3, further studies are required to clarify this issue.

Taken together, we propose that NANOS3 functions in spermatogonial progenitors to prevent their premature differentiation by inhibiting the RA signaling pathway. Furthermore, we suggest that NANOS3-mediated inhibition of spermatogonial differentiation is important to initiate synchronized spermatogenesis from seminiferous stages VII-VIII, and indirectly helps to maintain correct testicular germ cell associations. Further molecular analysis is necessary to clarify how NANOS3 represses premature differentiation. There are only a few reported factors that regulate spermatogonial progenitors (Chakraborty et al., 2014; Matson et al., 2010; Shinoda et al., 2013; Zhang et al., 2016). Thus, this study provides a key to understanding the mechanisms that underlie the maintenance of the progenitor state.

## MATERIALS AND METHODS

### Mice

All mice were bred and maintained in the animal facility at the National Institute of Genetics. Wild-type mice were obtained from CLEA, Japan. Nanos3-L-pA (Nanos3+/L), Nanos3-Cre (Nanos3+/Cre) and CAG-CAT-EGFP reporter mice were maintained and used as previously described (Sada et al., 2009; Suzuki et al., 2008; Tsuda et al., 2006). *Ngn3-GFP* and endo-cKO mice used in this study were described previously (Wright et al., 2020; Yoshida et al., 2004). All mice used in this study are of a mixed background (C57BL6/N, C3H and ICR). All experiments were conducted in accordance with the guidelines of the National Institute of Genetics. Genotypes were determined by PCR using genomic DNA isolated from tails. The primers are listed in Table S4.

### Generation of transgenic mice

*BAC-floxed-Nanos3-Rfp* and *CAG-floxed-mRFP-3xFlag-Nanos3* were generated by microinjection of the constructs into one-cell-stage fertilized eggs (C57BL6/N×C3H F1). Injected eggs were cultured until the two-cell stage and then transferred into the oviducts of pseudopregnant foster mothers.

### Epididymal sperm counts

The cauda epididymis was dissected and minced in 2 ml of PBS. Sperm-containing PBS was filtered through a 100-μm cell strainer (BD Falcon) to remove tissue fragments. Filtered PBS was diluted 1:40 with PBS and the sperm were counted with a hemocytometer.

### Histological analysis

Testes and epididymides were fixed in Bouin's solution or 4% PFA overnight at 4°C and then embedded in paraffin or OCT compound. The paraffin or frozen sections (6 μm) were stained with hematoxylin and eosin (Sakura Finetek Japan). The stained sections were mounted with Mount-Quick (Daido Sangyo) and observed using an Olympus DP80 microscope.

### Immunofluorescence staining of cross-sections

Testes and embryonic gonads were fixed in 4% PFA overnight at 4°C and embedded in OCT compound (Tissue Tek, Sakura) or paraffin. The frozen or paraffin sections (6 μm) were blocked with 3% skim milk (Wako) for 1 h at room temperature (RT). Thereafter, the sections were incubated with primary antibodies overnight at 4°C. After washing with PBS containing 0.1% Tween (0.1% PBS-T), sections were incubated with secondary antibodies for 1 h at RT. The sections were washed with 0.1% PBS-T and then counterstained with DAPI (Sigma-Aldrich). After washing, these sections were mounted and observed using an Olympus FV1200 confocal microscope.

Primary antibodies were used at the following dilutions: chicken anti-GFP (1:500, Aves), rabbit anti-PLZF (1:200, Santa Cruz Biotechnology), goat anti-GATA4 (1:100, Santa Cruz Biotechnology), goat anti-GFRA1 (1:200, R&D), rabbit anti-SOX9 (1:100, Santa Cruz Biotechnology), rabbit anti-SYCP3 (1:200, Abcam), Armenian hamster anti-KIT (1:100, a gift from Dr T. Hirata), rabbit anti-phosphorylated histone H3 (1:100, Millipore), goat anti-CDH1 (1:200, R&D), mouse anti-FLAG (1:5000, Sigma-Aldrich), anti-NANOS3 (1:100) (Suzuki et al., 2007), rat anti-STRA8 (1/3000, a gift from Dr K. Ishiguro), rabbit anti-cleaved PARP (1:200, Cell Signaling Technology), rat anti-GATA1 (1:200, Santa Cruz Biotechnology), rabbit anti-SYCP1 (1:200, Abcam), mouse anti-DMRT1 (1:200, Santa Cruz Biotechnology), rat anti-TRA98 (1:1000, BioAcademia), rabbit anti-RARγ (1:200, Cell Signaling Technology) and rabbit anti-NANOS2 (1:200) (Suzuki et al., 2007). All secondary antibodies, anti-rabbit, anti-goat, anti-mouse, anti-rat or anti-chick IgG antibodies conjugated with either Alexa-488 or Alexa-594 (1:500, Invitrogen) and anti-Armenian Hamster IgG conjugated with Cy3 (Jackson ImmunoResearch), were used at a dilution of 1:500.

### Classification of seminiferous tubule stage

Seminiferous tubule stages were classified by nuclear morphology based on Russell et al. (Russell et al., 1990). In addition, the detailed staging was confirmed by the expression patterns of STRA8 and SYCP1. Briefly, STRA8 should be detected from undifferentiated spermatogonia of stage VIII to typeA differentiated spermatogonia of stage XII and from late pre-leptotene spermatocytes of stage VII to early leptotene spermatocytes of stage VIII. SYCP1 should be detected from zygotene spermatocytes of stage XI to late-pachytene spermatocytes of stage X and be especially strongly detected from late-zygotene spermatocytes of stage XII to mid-pachytene spermatocytes of stage VII.

### Wholemount immunostaining

Dissected seminiferous tubules were fixed in 4% PFA for 4 h at 4°C and attached to MAS-coated slides (Matsunami). The samples were dehydrated with a methanol series [25, 50, 75 and 100% in PBS containing 0.1% Tween (0.1% PBS-T)]. After dehydration, they were blocked in 3% BSA (Sigma-Aldrich) containing 0.1% PBS-T for 1 h at RT. The samples were then incubated with primary antibodies overnight at 4°C. After washing with 0.1% PBS-T, they were incubated with secondary antibodies for 1 h at RT. The samples were washed with 0.1% PBS-T and then counterstained with DAPI (Sigma-Aldrich). After washing, these samples were mounted and observed using an Olympus FV1200 confocal microscope.

Primary antibodies were used at the following dilutions: rat anti-GFP (1:500, Nacalai Tesque), goat anti-GFRA1 (1:200, R&D), Armenian hamster anti-KIT (1:100, a gift from Dr T. Hirata), goat anti-CDH1 (1:200, R&D), rabbit anti-RFP (1:200, Rockland) and rat anti-STRA8 (1/3000, a gift from Dr K. Ishiguro) (Ishiguro et al., 2020). All secondary antibodies, anti-rabbit, anti-goat or anti-rat IgG antibodies conjugated with either Alexa-488 or Alexa-594 (1:500, Invitrogen) and anti-Armenian Hamster IgG conjugated with Cy3 (Jackson ImmunoResearch), were used at a dilution of 1:250.

### Statistics

The Shapiro-Wilk test was used to assess the normality in frequency. The *F* test was used to compare the standard deviation. The Student's *t*-test was used for statistical comparison in this study. The significance level was set at *P*<0.05.

### WIN18,446 and RA treatment

At 8.6 days before 12 weeks of age (day -8.6) endo-cKO and control mice received i.p. injections of 33 mg/kg of all-trans RA (10% DMSO/90% corn oil, Fujifilm-Wako) (day -8.6). From the day after RA injection, the mice received i.p. injections of 100 mg/kg/day of WIN18,446 (5% DMSO/95% corn oil, Cayman Chemical) for 8 days (from day -8 to day -1). On the day of 12 weeks of age (day 0), the right testis was collected from the mice and the second RA injection was administered. Then, the left testis was collected on day 1, 5 or 10. The WIN7D+RA method is as previously descripted (Beedle et al., 2019; Endo et al., 2017; Hogarth et al., 2013). Briefly, P2 male mice received s.c. injections of 1 mg/mouse/day of WIN18,446 for 7 days (from P2 to P8). On day P9, the mice received i.p. injections of 0.25 mg/mouse of RA (10% DMSO/90% saline).

### RNA immunoprecipitation (IP) and RT-qPCR

IP and RT-qPCR were performed as previously described (Niimi et al., 2019). Briefly, the testes from *Nanos3*-OE mice were homogenized on ice in IP buffer and centrifuged at 10,000× ***g*** for 10 min at 4°C. Next, 5 M NaCl was added to the supernatants to a final concentration of 150 mM. The samples were mixed with anti-FLAG M2 affinity gel (A2220, Sigma-Aldrich, MO, USA) or mouse IgG-agarose (A0919, Sigma-Aldrich, MO, USA) and incubated with rotation for 3 h at 4°C. After five washes with IP buffer containing 150 mM NaCl, precipitates were eluted with 3×FLAG peptides (Sigma-Aldrich) and then co-precipitated RNAs were purified using TRIzol^®^ Reagent (Life Technologies, CA, USA).

The immunoprecipitated RNAs were then reverse transcribed by Superscript III reverse transcriptase (Invitrogen) and (dT)_20_ primer (Invitrogen). Quantitative PCR was performed on the Thermal Cycler Dice^®^ Real Time System (Takara Bio, Shiga, Japan) using SYBR^®^ *Premix Ex Taq™* II (Tli RNaseH plus) (Takara Bio) in 20-μl reactions. Primer pairs are described in Table S3. Each sample was analyzed in triplicate and three biological replicates (*n*=3) were included. The relative transcript amount was calculated by the standard curve method using Multiple RQ Software (Takara Bio). The level of *Actinβ* control mRNA was used to normalize mRNA levels of target genes. The fold enrichment of each mRNA in IP of anti-FLAG M2 affinity gel (α-FLAG, A2220, Sigma-Aldrich, MO, USA) compared with IP of Mouse IgG-Agarose (IgG, A0919, Sigma-Aldrich, MO, USA) was calculated (ratio of each mRNA level in FLAG IP to IgG IP).

### Western blotting

Samples were boiled in 2× sample buffer for 5 min and run on gels for sodium dodecyl sulphate-polyacrylamide gel electrophoresis (SDS-PAGE), and then electroblotted onto nitrocellulose membrane (BioTrace NT, Pall Corporation, USA). Membranes were blocked in 5% skim milk/PBST for 1 h at RT and then incubated with the following primary antibodies overnight at 4°C: rabbit anti-NANOS3 (1/800) (Suzuki et al., 2007) and rabbit anti-DND1 (1/1000) (Suzuki et al., 2016). After washing three times with PBST, membranes were incubated with HRP-conjugated secondary antibodies (1/10,000, sc-2054, Santa Cruz Biotechnology, USA) for 1 h at RT. Immunoreactivity was visualized as chemiluminescence using Western BLoT Chemiluminescence HRP Substrate (Takara Bio Inc., Japan) and a lumino-image analyzer (ImageQuant LAS-4000mini, GE Healthcare, England). All antibodies used in western blotting analysis were diluted in Can Get Signal Immunoreaction Enhancer Solution (NKB-101; Toyobo Co., Ltd.).

## Supplementary Material

Supplementary information
