# Supplementary material for: NANOS3 suppresses premature spermatogonial differentiation to expand progenitors and fine-tunes spermatogenesis in mice
Source: Biol Open. 2022 Apr 8;11(4):bio059146. doi: 10.1242/bio.059146 (PMC9002807; doi:10.1242/bio.059146)
Supplement: Supplementary information [file biolopen-11-059146-s1.pdf]

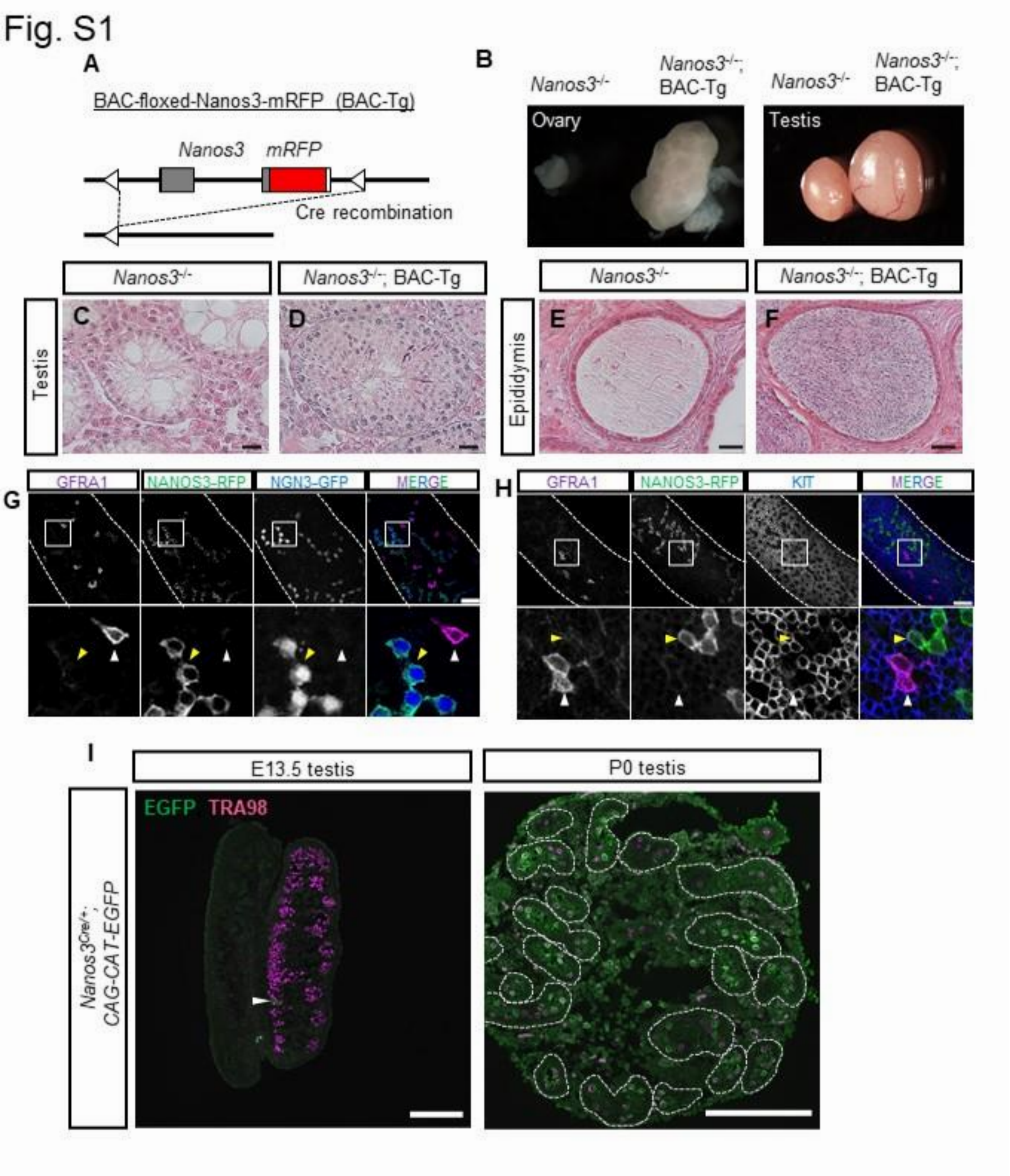

Fig. S1
A
BAC-floxed-Nanos3-mRFP (BAC-Tg)
Nanos3
mRFP
Cre recombination
B
Nanos3-/-
Nanos3-/-; BAC-Tg
Ovary
Testis
Testis
Nanos3-/-
Nanos3-/-; BAC-Tg
C
D
Epididymis
E
F
G
GFRA1
NANOS3-RFP
NGN3-GFP
MERGE
H
GFRA1
NANOS3-RFP
KIT
MERGE
I
E13.5 testis
P0 testis
Nanos3Cre/+; CAG-CAT-EGFP
EGFP
TRA98

Fig. S1. ***Nanos3*-deficient phenotype is rescued by the introduction of BAC-Tg.** (A) A schematic construct of the BAC transgene. The floxed *Rfp-tagged Nanos3* sequence is removed after Cre-mediated recombination.(B) Ovaries and testes from 8-week-old *Nanos3*$^{-/-}$ and *Nanos3*$^{-/-}$, BAC-Tg mice. Atrophy of the ovary and testis in *Nanos3*$^{-/-}$ was rescued by BAC-Tg introduction. (C to F) HE-stained cross-sections of testes and the epididymis. The germ cell-less phenotype was rescued in *Nanos3*$^{-/-}$, *BAC-Tg* mice (D, F). Scale bars = 20 μm (C, D), 50 μm (E, F). (G-H) Whole-mount immunostaining of seminiferous tubules from 8-week-old mice carrying BAC-Tg with anti-GFRA1, anti-RFP, anti-GFP and anti-KIT antibodies. The magnified images are shown below. White arrowheads indicate GFRA1-positive A_s or A_{pr} spermatogonia. Yellow arrowheads indicate NANOS3-RFP-positive spermatogonia. Scale bars = 50 μm. (I) Immunostaining of the embryonic testis from E13.5 and P0 mice carrying *Nanos3-Cre* and *CAG-CAT-EGFP* with anti-GFP and anti-TRA98 antibodies. The arrowheads indicate GFP-positive germ cells. Scale bars = 200 μm

Biology Open • Supplementary information

Fig. S2

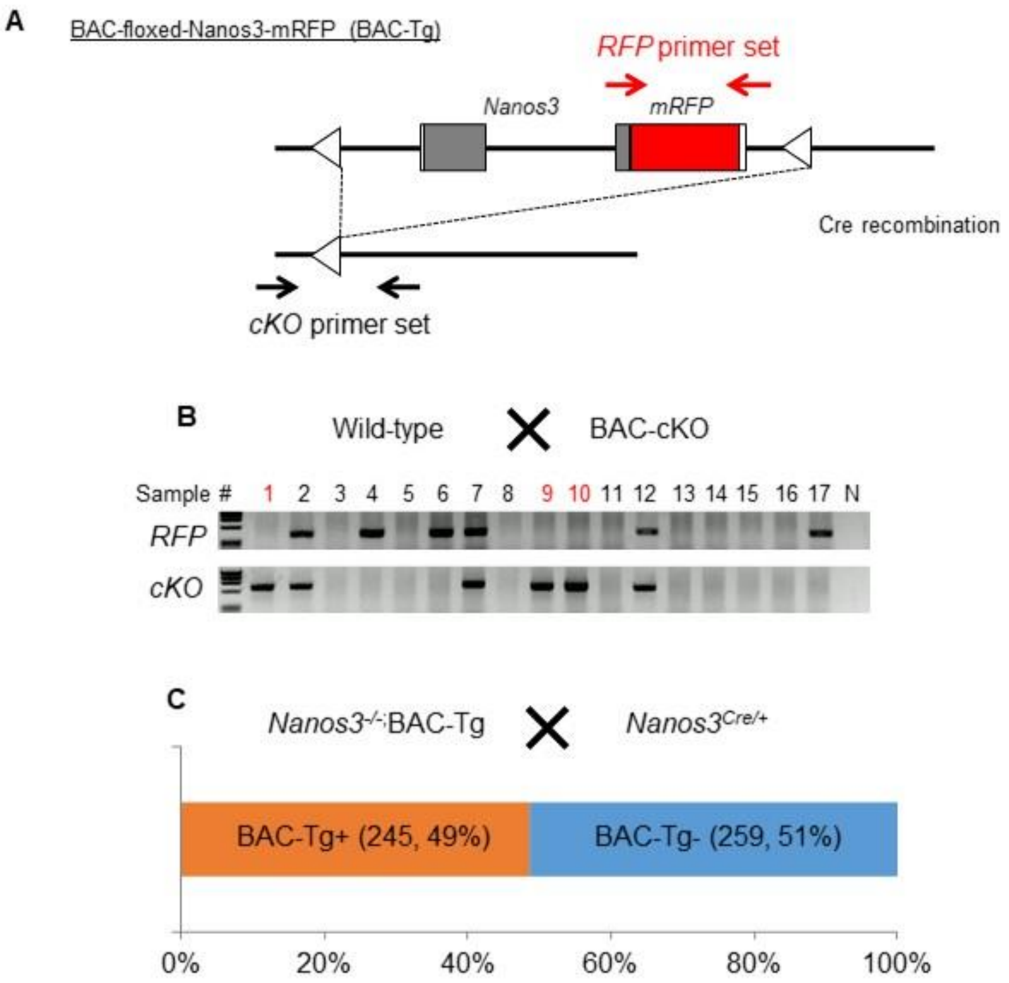

D

	A single - A aligned			A aligned		
RFP	nega	posi		nega	posi	
BAC-cKO#1	736	59	7.4%	188	32	14.5%
BAC-cKO#2	783	72	8.4%	217	39	15.2%
BAC-cKO#3	399	49	10.9%	110	28	20.3%
average			8.9%			16.7%

Fig. S2. BAC-cKO germ cells can differentiate into functional sperm.
(A) A schematic construct of the BAC-Tg and the primer sets for genotyping. Black arrows represent the location of primer pairs to detect the *Nanos3-Rfp*-deleted transgenic allele. The undeleted allele was detected by the primer pairs that amplify the *Rfp* sequence. (B) Genotyping of the offspring produced by crossing wild-type females with BAC-cKO males. Offspring #1, # 9 and #10 only had the *Nanos3-Rfp*-deleted allele. #2, 7 and 12 had both deleted and undeleted transgenes. N: negative control. (C) Genotyping results of the offspring (total 504 samples) produced by crossing *Nanos3*$^{-/-}$*; BAC-Tg* females with *Nanos3*$^{Cre/+}$ males. (D) Quantitation of RFP-negative and –positive undifferentiated spermatogonia clusters detected by whole-mount immunofluorescence for CDH1 and RFP (n=3).

Biology Open • Supplementary information

Fig. S3

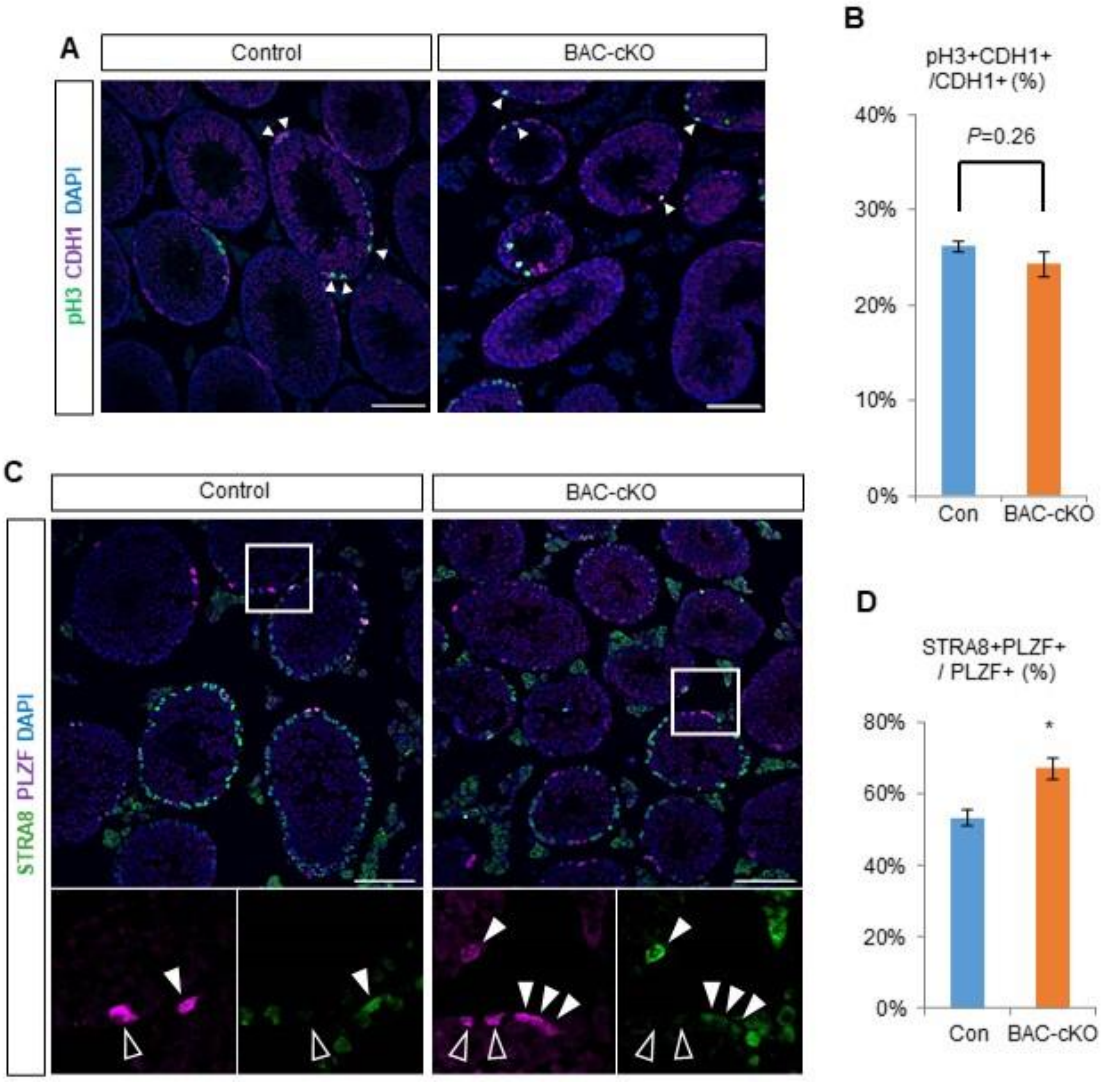

Fig. S3. STRA8 upregulation in spermatogonial progenitors in BAC-cKO mice. (A) Immunostaining of 8-week-old testes from BAC-cKO or control mice. The proliferating cell marker pH3 and undifferentiated spermatogonia marker CDH1 are shown in green and magenta, respectively. Nuclei were counterstained with DAPI (blue). pH3- and CDH1-double-positive cells are indicated by arrowheads. Scale bar = 100 μm. (B) Quantitative data of pH3-positive cells among CDH1-positive undifferentiated spermatogonia. There was no significant difference (*P*=0.26) between control and BAC-cKO mice. Values represent the mean ± SEM. (C) Immunostaining of adult testes. The marker of RA signal, STRA8, and undifferentiated spermatogonia marker PLZF are shown in green and magenta, respectively. Bottom panels show enlarged images. STRA8 and PLZF-double-positive differentiating spermatogonia and STRA8-negative, PLZF-positive undifferentiated spermatogonia are indicated by closed arrowheads and open arrowheads, respectively. Scale bar = 100 μm. (D) Quantitative data of STRA8-positive differentiating cells among PLZF-positive undifferentiated spermatogonia. Values represent the mean ± SEM. **P*<0.05.

Biology Open • Supplementary information

Fig. S4

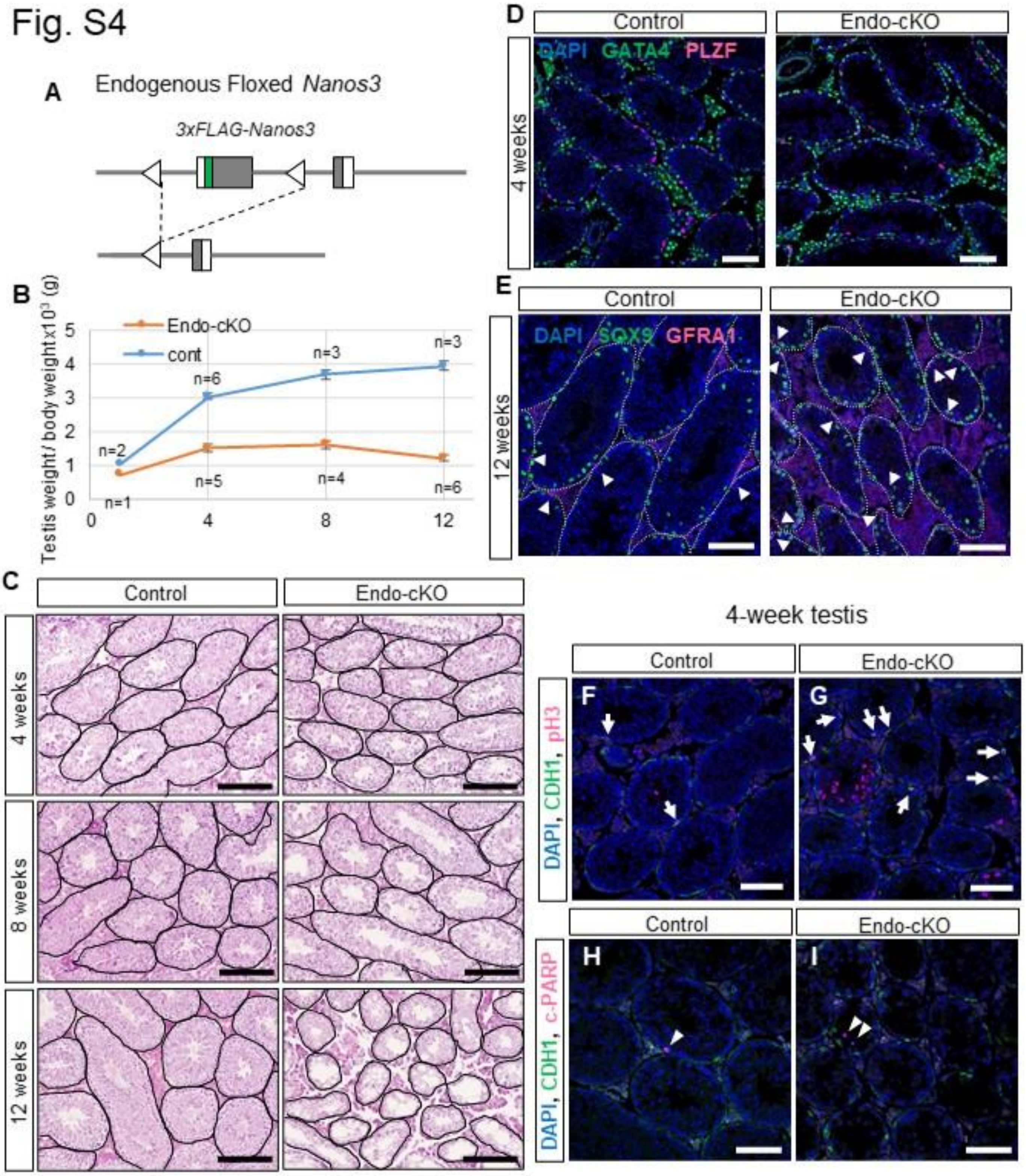

Biology Open • Supplementary information

Fig. S4. (A) A schematic drawing of the floxed endogenous *Nanos3* allele. The boxes indicate *Nanos3* exons. *3xFLAG* and the *Nanos3* coding region are indicated by green and grey, respectively. Lox sequences are indicated by triangles. (B) Testis weight to body weight ratio in 1, 4, 8 and 12-week-old mice. Values represent the mean ± SEM. (C) HE-stained testes from 4, 8 and 12-week-old control and endo-cKO mice. The seminiferous tubules are surrounded by black lines. Scale bar = 200 μm. (D) Immunostaining of 4-week-old testes from control and endo-cKO. PLZF and the Sertoli cell marker GATA4 are shown in magenta and green, respectively. Scale bar = 100 μm. (E) Immunostaining of 12-week-old testes from control and endo-cKO. GFRA1 and the Sertoli cell marker SOX9 are shown in magenta and green, respectively. GFRA1-positive cells are indicated by arrowheads. Scale bar = 100 μm. (F, G) CDH1 and phosphorylated histone H3 (pH3) signals are shown in green and magenta, respectively. Proliferating undifferentiated spermatogonia (pH3 and CDH1-double-positive cells) are indicated by arrows. Nuclei were counterstained with DAPI (blue). Scale bar = 100 μm. (H, I) CDH1 and the apoptotic cell marker cleaved PARP (c-PARP) signals are shown in green and magenta, respectively. C-PARP-positive cells are indicated by arrowheads. All c-PARP-positive cells were CDH1-negative. Nuclei were counterstained with DAPI (blue). Scale bar = 100 μm.

Biology Open • Supplementary information

Fig. S5

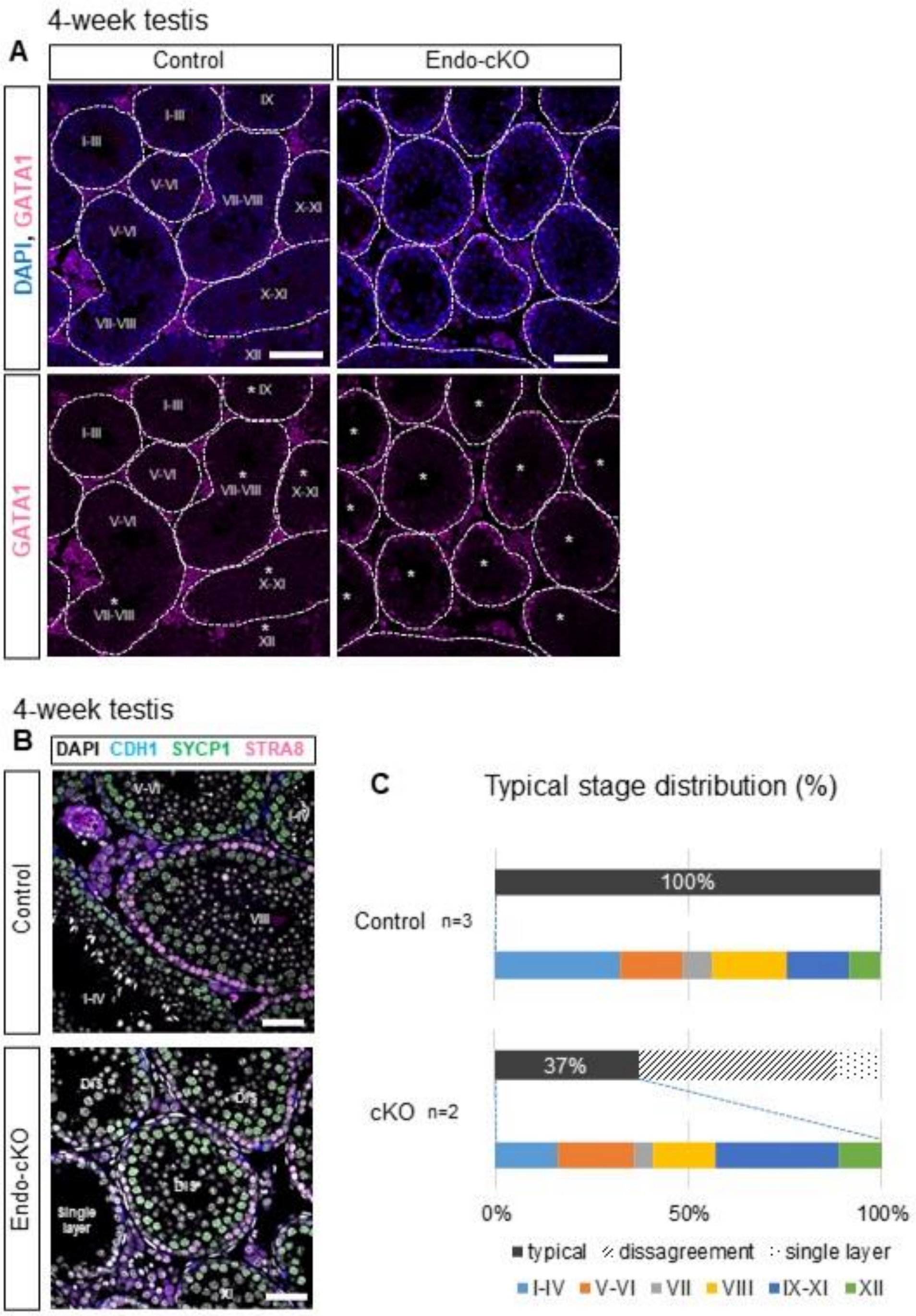

Fig. S5. (A) Immunostaining of 4-week-old testes with anti-CDH1 (green) and anti-GATA1 (magenta) antibodies. GATA1 is a stage VII-XII-specific Sertoli cell marker. GATA1 single-color images are shown on the bottom. White dotted lines indicate the basal membrane of seminiferous tubules. Seminiferous tubule stages are indicated by Roman numerals in control images. The tubules with strong GATA1 signals are indicated by asterisks. Scale bar = 100 µm. (B) Immunostaining of 4-week-old testes with anti-CDH1, anti-STRA8 and anti-SYCP1. Seminiferous stages are indicated in Roman numerals. Tubules containing atypical combinations of spermatogenic cells are labeled as “Dis” (Disagreement). Scale bar = 50 µm. (C) The percentage of tubules exhibiting typical or abnormal germ cell associations in the control and endo-cKO. Seminiferous stage distribution in tubules exhibiting typical germ cell associations are also shown in separate columns.

Biology Open • Supplementary information

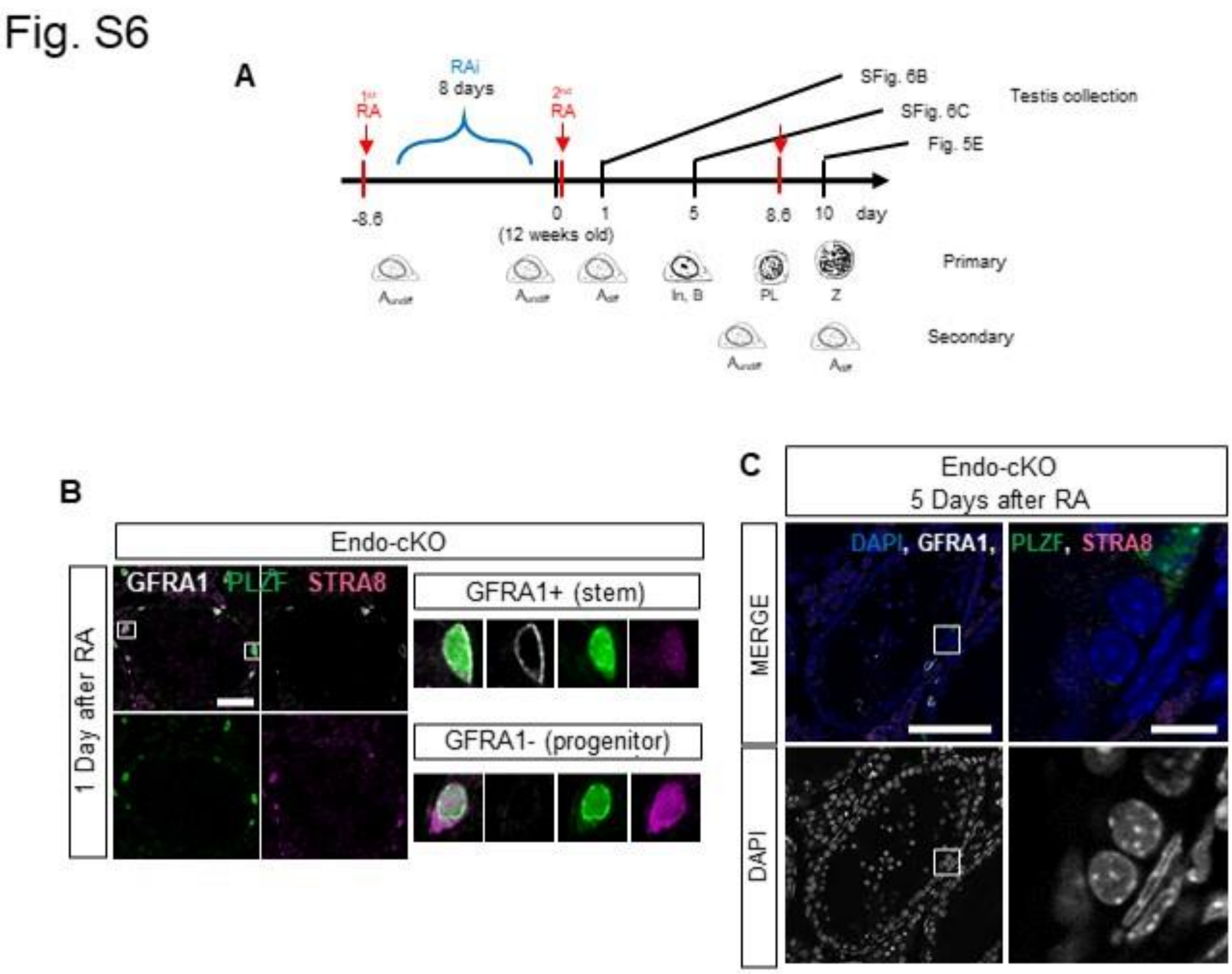

Fig. S6. (A) Scheme of the experiment. RA was injected 8.6 days before the day of 12 weeks of age, followed by daily RAi injection for 8 days. One of two testes was collected on the day of 12 weeks of age and then RA was injected again. At 1, 5 or 10 days after the second RA injection, the other testis was collected. The expected stage in the synchronized tubules of "the first wave" and "the second wave" is shown below. (B) Immunostaining of testes 1 day after the second RA injection. GFRA1, PLZF and STRA8 are shown in white, green and magenta, respectively. Magnified images of GFRA1-positive stem cells and GFRA1-negative progenitor cells are shown in the right panels. Scale bar = 50 µm. (C) Immunostaining of testes 5 days after the second RA injection. GFRA1, PLZF and STRA8 are shown in white, green and magenta, respectively. Nuclei were counterstained with DAPI (blue). Magnified images are shown in the right panels. Scale bar = 100 µm or 10 µm (in magnified image of C).

Biology Open • Supplementary information

Fig. S7

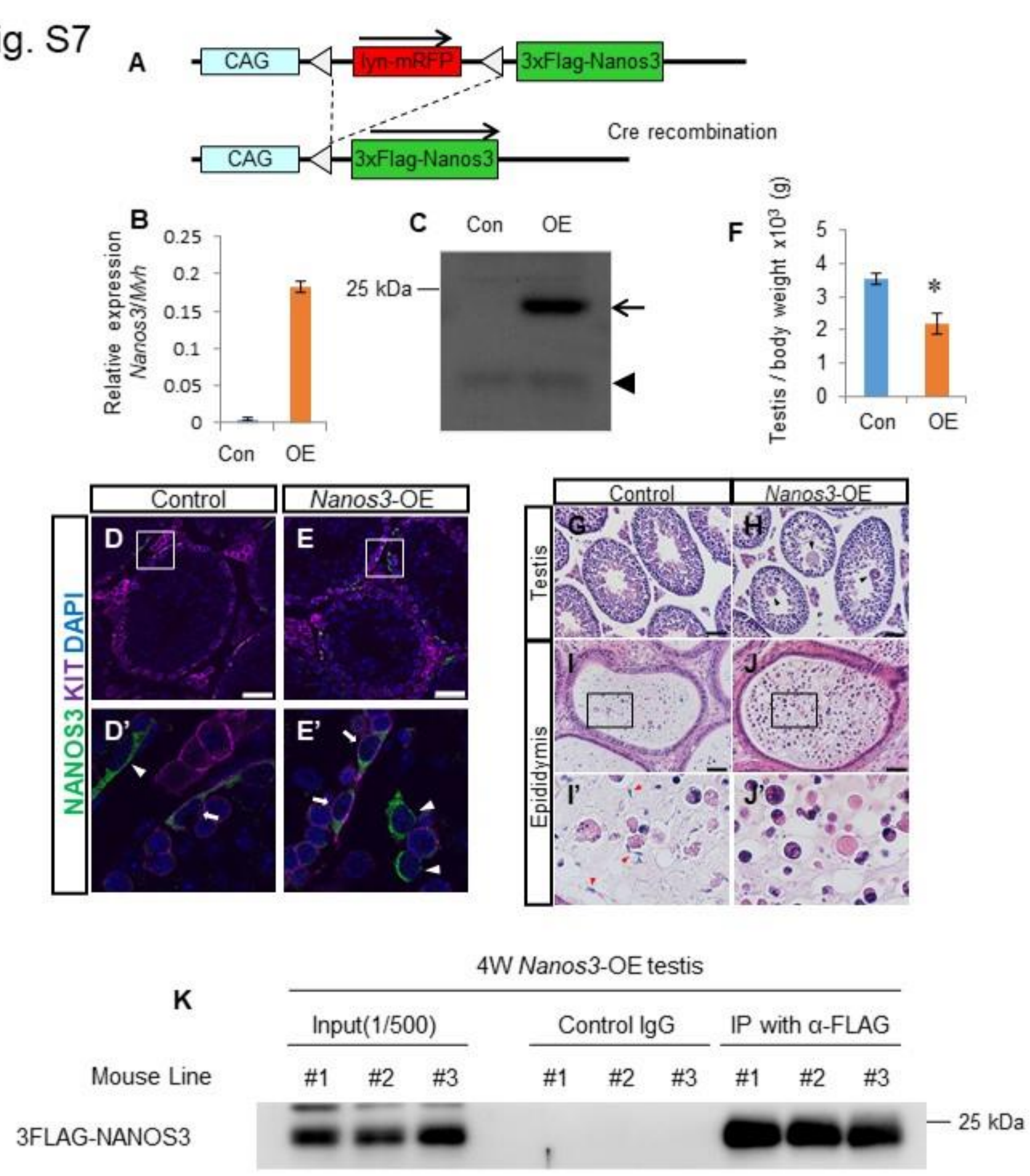

Biology Open • Supplementary information

Fig. S7. Testicular abnormalities observed in *Nanos3*-OE mice. (A) A schematic of the transgene *CAG-floxed-mRFP-3xFlag-Nanos3*. Nanos3 was induced by Cre recombination. (B) Quantification of *Nanos3* mRNA in 3W control and Nanos3-OE testes using qPCR. Mvh, a germ cell-specific gene, was used for normalization. Values represent the mean ± SD. (C) Western blotting of 3W testes with anti-NANOS3 antibody. Transgene-derived 3xFLAG-NANOS3 and endogenous NANOS3 bands are indicated by an arrow and arrowhead, respectively. (D-E) Immunostaining of 4W testes from control and *Nanos3*-OE mice with anti-NANOS3 (green) and anti-KIT (magenta) antibodies. Nuclei were counterstained with DAPI (blue). Magnified images are shown in D' and E'. Scale bar = 50 μm. (F) Testis weight to body weight ratio in 6W mice. Values represent the mean ± SEM. *P<0.05. (G-J) HE-stained 4W testes (G, H) and 6W epididymides (I, J) from control and *Nanos3*-OE mice. Magnified images of epididymides are shown in I' and J'. Abnormal cell clumps are indicated by black arrowheads (H). Spermatozoa in the epididymis are indicated by red arrowheads (I'). Scale bar = 50 μm. (K) Western blot analyses of proteins co-precipitated with anti-FLAG antibody from testis extracts of 4-week-old *Nanos3*-OE mice. IgG was used as a negative control.

Fig. S8

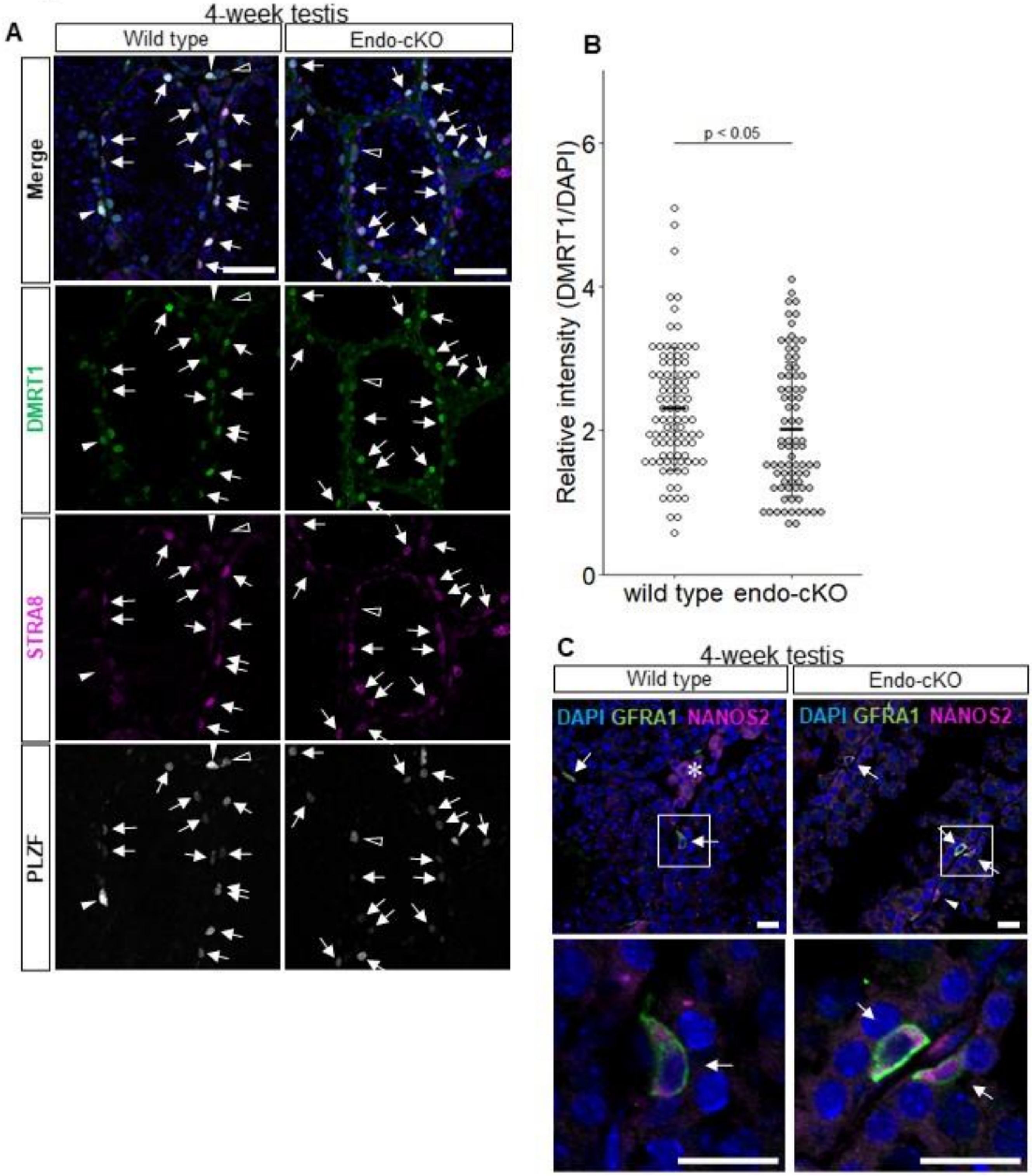
A
4-week testis
Wild type
Endo-cKO
Merge
DMRT1
STRA8
PLZF
B
p < 0.05
Relative intensity (DMRT1/DAPI)
0
2
4
6
wild type
endo-cKO
C
4-week testis
Wild type
Endo-cKO
DAPI GFRA1 NANOS2
DAPI GFRA1 NANOS2

Fig. S8. DMRT1 expression level was decreased in *Nanos3*-cKO mice.
(A) Immunostaining of 4-week-old testes of wild-type and endo-*Nanos3*-cKO mice with anti-DMRT1 (green), anti-STRA8 (magenta) and anti-PLZF (grey) antibodies. Arrows indicate the PLZF and STRA8-positive differentiating spermatogonia. White arrowheads indicate the STRA8-negative PLZF-positive undifferentiated spermatogonia. Open arrowheads indicate the DMRT1-negative PLZF-positive undifferentiated spermatogonia. Scale bar = 50 µm. (B) Relative signal intensity of DMRT1 normalized by DAPI signal intensity. Values represent the mean ± SD. (C) Immunostaining of 4-week-old testes with anti-GFRA1 (green) and anti-NANOS2 (magenta) antibodies. The magnified images are presented below. Arrows indicate the NANOS2 and GFRA1-double-positive spermatogonia. Arrowheads indicate the NANOS2-positive GFRA1-negative spermatogonia. Asterisks indicate the non-specific signals in the interstitial cells. Scale bar = 20 µm.

Biology Open • Supplementary information

Fig. S9

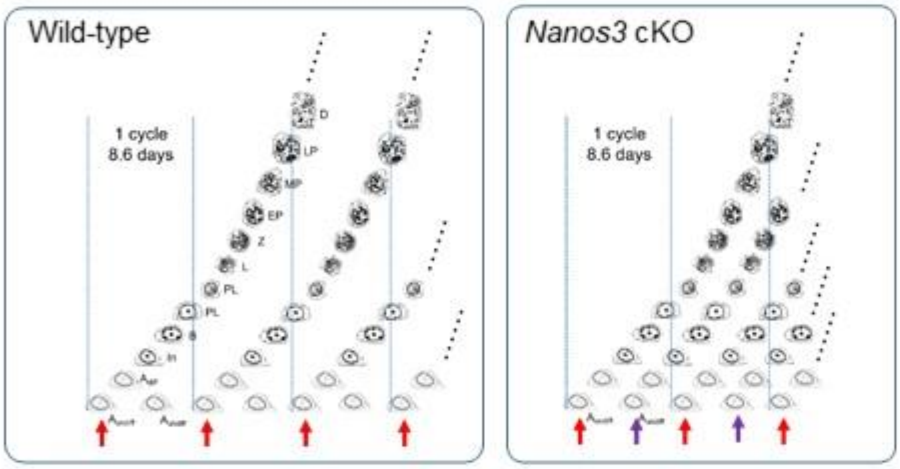

Fig. S9. The hypothesized model of the *Nanos3* cKO phenotype. Interpretation of the results. In the wild-type, differentiation is induced only when undifferentiated spermatogonia receive RA signals every 8.6 days (indicated by red arrows), whereas in the endo-cKO, undifferentiated spermatogonia prematurely differentiate (indicated by purple arrows), which results in acceleration of the cycle and generates disagreement within a tubule. The illustrations are from Russell et al. and Endo et al. (Endo et al., 2017; Russell et al., 1990).

Fig. S10

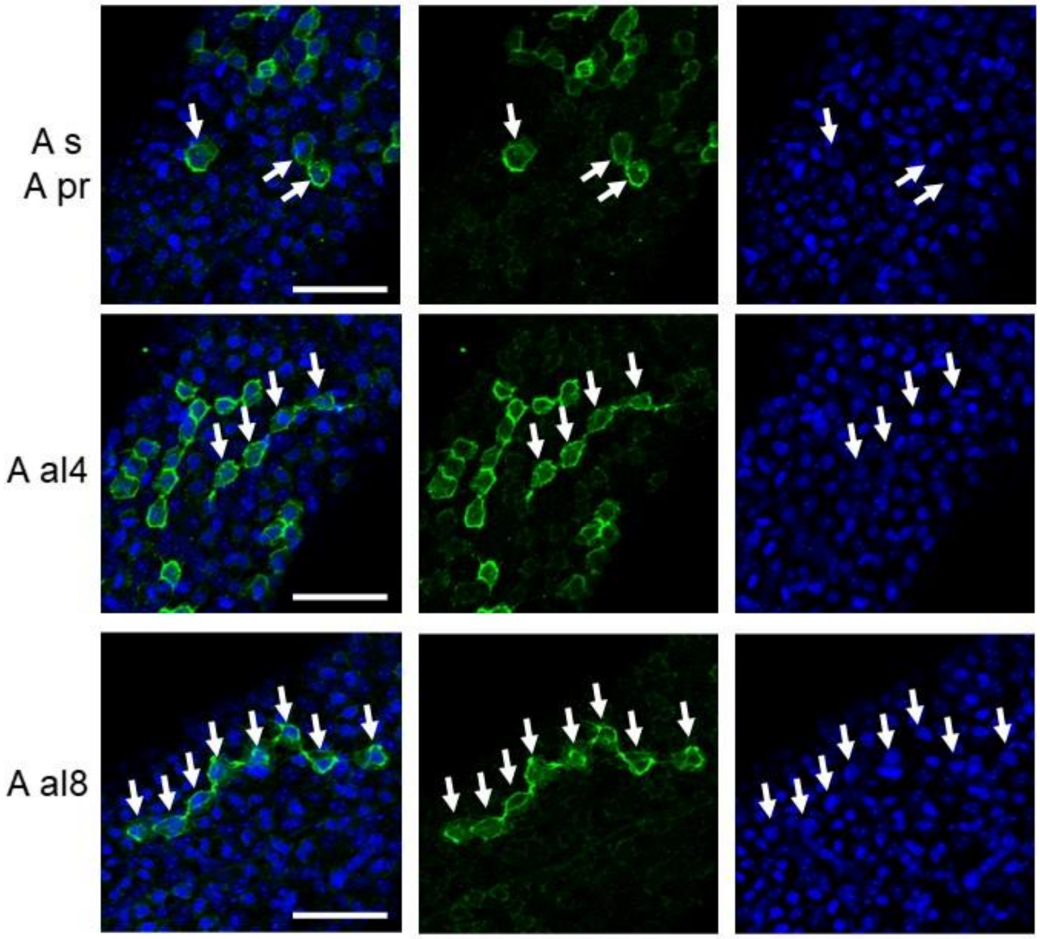

Fig. S10. The images of undifferentiated spermatogonial clusters. Whole-mount immunostaining of seminiferous tubules from adult mice carrying BAC-Tg with anti-CDH1 antibodies (green). Nuclei were counter-stained with DAPI (blue). White arrows indicate CDH1-positive A_s, A_{pr}, A_{al4} or A_{al8} spermatogonia. Scale bars = 50 μm.

Biology Open • Supplementary information

Table S1. Seminiferous stage distribution in Rai-treated testes

		stage												tubule number	n
		I-IV		V-VI		VII-IX		X-XII		disagreement		single layer			
RAi	cont	41	(37%)	15	(14%)	30	(27%)	24	(22%)	0	(0%)	0	(0%)	110	3
	cko	11	(15%)	3	(4%)	11	(15%)	31	(43%)	10	(14%)	7	(10%)	72	2
Day 10	cont	2	(3%)	0	(0%)	9	(11%)	59	(75%)	6	(8%)	3	(4%)	79	1
	cKO	0	(0%)	0	(0%)	0	(0%)	65	(74%)	20	(23%)	3	(3%)	88	1

Table S2. Spermatogenic stage distribution in the synchronized tubules at stages X-XII.

	spermatogenic stage				
	undiff	A	In	B	pL
cont	68	55	10	0	0
	(51%)	(41%)	(8%)	(0%)	(0%)
cko	167	163	60	27	5
	(40%)	(39%)	(14%)	(6%)	(1%)

Biology Open • Supplementary information

Table S3. The proportion of DMRT1- and STRA8-positive cells among PLZF-positive undifferentiated spermatogonia.

	PLZF +										
	DMRT1 -						DMRT1 +				
	STRA8 -			STRA8 +			STRA8 -			STRA8 +	
wild type (189 cells)	41	22%		1	1%		75	40%		72	38%
endo-cKO (174 cells)	34	20%		0	0%		55	32%		85	49%

Table S4. PCR primers.

Genotyping	Forward	Reverse
Nanos3 WT	5'- CCAGCCATGGGGACTTTC-3'	5'- GGGACTGATAGATGGCAC-3'
Nanos3-lacZ knockin	5'- ACTATCCCGACCGCCTTACT-3'	5'- GGGACTGATAGATGGCAC-3'
BAC-Tg (*mRFP*)	5'- AAGCTGAAGGTGACCAAGGG-3'	5'- GTAGGTGGTCTTGACCTCGG-3'
Nanos3-Rfp deleted	5'-GTAACCTTGAGGCTGCTTAGCT-3'	5'-GCAGATGGGTAATGAGCGAGC-3'
Nanos3-Cre	5'-GGACATGTTCAGGGATCGCCAGGCG-3'	5'-GCATAACCAGTGAAACAGCATTGCTG-3'
GFP	5'-CCTGGTCGAGCTGGACGGCGAC-3'	5'-TCACGAACTCCAGCAGGACCATG-3'
Nanos3 flox	5'-CTTTCAGCCCACACAAACACCATATA-3'	5'-GACACACATAATCCCGCAAAATGG-3'
Nanos3 flox deleted	5'-CTTTCAGCCCACACAAACACCATATA-3'	5'-CTGCTAGAAGTCCCCGTCTCTGG-3'
qPCR	Forward	Reverse
Actin β (NM_007393.5)	5'-AAAGACCTCTATGCCAACAC-3'	5'-TGCTTGCTGATCCACATCTG-3'
Sohlh1 (NM_001001714.1)	5'-AGCCAGACTCCGGTATAGCCA-3'	5´-CAAGCTGGAAGACTCTGGCT-3´
Sohlh2 (NM_028937.3)	5'-CTTTGGAGGGAGCAGTGAGAG-3'	5´-GTGCAGTGGGTGGCAAATAAG-3´
Dmrt1 (NM_015826.5)	5'-CTGGAAACCAGTGGCAGATG-3'	5´-GCGAGAACACACTGGCTTTG-3´
Dazl (NM_010021.5)	5'-CTATTCTGTCAGGATTGCTC-3'	5´-CAGTTGTGATATGACCCATT-3´
Taf7l (NM_028958.4)	5'-GAGGGACAGAAGTATGTGGT-3'	5'-TTTAGCCTCCATGAAGCAGA-3'
Mvh (*Ddx4*) (NM_001145885.1)	5'-GCTTCATCAGATATTGGCGAGT-3'	5´-GCTTGGAAAACCCTCTGCTT-3´
Nanos3 (NM_194059.2)	5'- GAGTCCCGTGCCATCTATCAG-3'	5'- GCTGGTGAGTGGGCAAAAG-3'

Biology Open • Supplementary information
